# *Tbx4* function during hindlimb development reveals a mechanism that explains the origins of proximal limb defects

**DOI:** 10.1242/dev.199580

**Published:** 2021-09-24

**Authors:** Veronique Duboc, Fatima A. Sulaiman, Eleanor Feneck, Anna Kucharska, Donald Bell, Muriel Holder-Espinasse, Malcolm P. O. Logan

**Affiliations:** 1 Randall Centre for Cell and Molecular Biophysics, King's College London, Guy's Campus, London SE1 1UL, UK; 2 Light Microscopy, Francis Crick Institute, 1 Midland Road, London NW1 1AT, UK; 3 Clinical Genetics Department, Guy's Hospital, London SE1 9RT, UK

**Keywords:** Congenital limb abnormalities, Chondrogenesis, Limb formation, *Tbx4*, *Pitx1*, Mouse

## Abstract

We dissect genetically a gene regulatory network that involves the transcription factors *Tbx4*, *Pitx1* and *Isl1* acting cooperatively to establish the hindlimb bud, and identify key differences in the pathways that initiate formation of the hindlimb and forelimb. Using live image analysis of murine limb mesenchyme cells undergoing chondrogenesis in micromass culture, we distinguish a series of changes in cellular behaviours and cohesiveness that are required for chondrogenic precursors to undergo differentiation. Furthermore, we provide evidence that the proximal hindlimb defects observed in *Tbx4* mutant mice result from a failure in the early differentiation step of chondroprogenitors into chondrocytes, providing an explanation for the origins of proximally biased limb defects.

## Abstract (precis)

**Summary:** Examination of the effects of loss of *Tbx4* reveals molecular and cellular mechanisms underlying proximally biased limb defects, which could explain the origins of the congenital limb abnormality, phocomelia.

## INTRODUCTION

Forelimbs and hindlimbs arise as outgrowths from the lateral plate mesoderm and form at fixed positions along the body axis. Forelimbs form at the cervical/thoracic junction whereas hindlimbs form at the lumbar/sacral junction and this position is conserved in vertebrates even when the number of segments/vertebrae differs between different species (Burke et al., 1995; Nishimoto and Logan, 2016). Initiation of forelimb and hindlimb formation starts with the expression of one of the T-box transcription factors *Tbx5* or *Tbx4* in restricted regions of the future forelimb- and hindlimb-forming lateral plate mesoderm, respectively. A key function of *Tbx5* and *Tbx4* is to activate *Fgf10* expression and establish an FGF signalling, positive-feedback loop that drives limb bud outgrowth (Nishimoto and Logan, 2016). This feedback loop is required for limb bud outgrowth and *Fgf10* mutant mice lack both forelimbs and hindlimbs (Sekine et al., 1999). Therefore, despite *Tbx4* and its paralogue *Tbx5* having dramatic hindlimb- and forelimb-restricted expression patterns, respectively (Logan et al., 1998; Rallis et al., 2003), both appear to have common roles in establishing limb bud formation by activating and establishing expression of *Fgf10* (Minguillon et al., 2005; Nishimoto and Logan, 2016; Nishimoto et al., 2015).

There are important differences, however, in how the forelimb and hindlimb buds are established. In the forelimb, *Tbx5* input is essential for *Fgf10* expression, whereas in the *Tbx4* mutant hindlimb some *Fgf10* expression is initiated and a small hindlimb bud forms (Naiche and Papaioannou, 2003). Therefore, although *Tbx4* is necessary for normal hindlimb *Fgf10* expression there is not the same exclusive requirement for *Tbx4* activity in the hindlimb to establish *Fgf10* expression as there is for its paralogue, *Tbx5*, in the forelimb. Additional inputs from other genes, such as *Isl1* (*Islet1*), appear to be required for the establishment of normal *Fgf10* expression in the hindlimb (Itou et al., 2012; Kawakami et al., 2011; Narkis et al., 2012).

To explore the differences in the pathways that establish the hindlimb bud, we have generated a complete loss of function of *Tbx4* in the hindlimb using a cre-deleter line, *RetRV5Cre*, which drives expression of cre recombinase throughout the hindlimb bud mesenchyme prior to hindlimb bud initiation. We demonstrate that there are significant differences between the forelimb and hindlimb initiation processes. Unlike in the forelimb, a dual input from *Tbx4* and the paired homeodomain transcription factor *Pitx1* are necessary to initiate *Fgf10* expression and drive further growth of the hindlimb bud. Furthermore, we clarify the epistatic relationship between *Tbx4*, *Pitx1* and *Isl1* during hindlimb budding, by showing that *Isl1* acts in parallel to *Pitx1* and *Tbx4* to initiate *Fgf10* expression. This distinction between forelimb and hindlimb developmental processes reveals an unexpected role for *Tbx4* during the formation of proximal skeletal elements.

We further demonstrate, using a gene deletion/gene replacement strategy, that in the absence of *Tbx4* expression, *Tbx5* can fully rescue hindlimb formation, further demonstrating that both paralogous genes exert an identical function during the development of forelimb and hindlimb. Furthermore, using an inducible *Fgf10* transgenic mutant, *Z/EGFgf10*, we show that the proximal bias of the phenotype observed in the *Tbx4* conditional mutant cannot solely be explained by the reduction of Fgf10 ligand expression. Finally, we provide evidence that the proximal hindlimb defects in the *Tbx4* mutant do not arise as a consequence of incorrect proximo-distal patterning, absence of *Fgf8* expression or cell death, but from a failure in the early differentiation step of chondroprogenitors into chondrocytes.

## RESULTS

### Conditional deletion of *Tbx4* produces proximally biased hindlimb defects

**Fig. 1.**
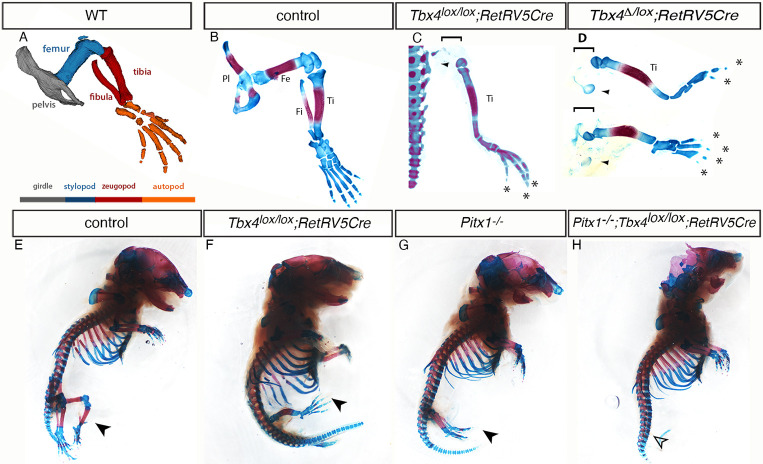
**Conditional deletion of *Tbx4* in the hindlimb results in absence of proximal elements and some digits.** (A) Schematic depicting the three anatomical regions of the limb – stylopod (blue), zeugopod (red) and autopod (orange) – and their composite skeletal elements. (B-D) Alcian Blue/Alizarin Red skeletal preparations of wild-type control (B; *n*=7), *Tbx4^lox/lox^;RetRV5Cre* (C; *n*=7) and *Tbx4*^Δ*/lox*^*;RetRV5Cre* (D; *n*=3) E17.5 hindlimbs. Arrowheads point to rudimentary pelvises; brackets show the absence of the femur; asterisks indicate the remaining digits. (E-H) *Tbx4* and *Pitx1* are required for hindlimb formation*.* Alcian Blue/Alizarin Red skeletal preparations of E17.5 control wild-type (E; *n*=7), *Tbx4^lox/lox^;RetRV5Cre* (F; *n*=7), *Pitx1^−/−^* (G; *n*=7) and *Pitx1^−/−^*;*Tbx4^lox/lox^;RetRV5Cre* (H; *n*=2) embryos. Black arrowheads indicate the hindlimbs; unfilled arrowhead indicates the absence of a hindlimb in H. Fe, femur; Fi, fibula; Pl, pelvis; Ti, tibula; WT, wild type.

The function of *Tbx4* in the hindlimb has been assessed previously by conditional deletion, using a constitutively active Cre and the limb-restricted *Prrx1Cre* transgene (Naiche and Papaioannou, 2003, 2007). Interpretation of the role of *Tbx4* in hindlimb initiation has proven difficult, however, because broad deletion of *Tbx4* is embryonic lethal at early limb bud stages and the *Prrx1Cre* transgene is active in the hindlimb bud only after it has formed (Logan et al., 2002). Following constitutive deletion of *Tbx4*, *Fgf10* expression in the nascent hindlimb-forming region is reduced and a small hindlimb bud forms (Naiche and Papaioannou, 2003). This is in marked contrast to the forelimb where conditional deletion of *Tbx5* leads to a failure to establish *Fgf10* expression and, as a consequence, complete absence of the forelimb bud. To conditionally delete *Tbx4* in the hindlimb-forming region, we generated a cre deleter transgenic mouse using a regulatory element from the *Ret* gene (Sukumaran et al., 2001). The activity of this isolated regulatory fragment does not replicate endogenous *Ret* expression from the intact locus. The *RetRVCre* line produces robust cre activity in the hindlimb-forming region prior to hindlimb bud formation (Fig. S1) and, significantly, this restricted expression does not generate the chorio-allantoic fusion defects observed in the constitutive *Tbx4* mutant (Naiche and Papaioannou, 2003). The *RetRVCre* deleter is therefore able to effectively conditionally delete *Tbx4* in the cells that normally give rise to the hindlimb without producing an early embryonic lethal phenotype (see below). We produced *Tbx4* mutant embryos that were either homozygous for the *Tbx4* conditional allele (*Tbx4^lox/lox^;RetRVCre*) (Fig. 1C) or carried one conditional allele and one deleted allele (*Tbx4*^Δ*/lox*^*;RetRVCre*) (Fig. 1D). In both cases, mutant embryos at embryonic day (E) 17.5 formed a small, rudimentary hindlimb comprising a tibia, a few malformed metatarsal and tarsal elements and two to four digits in the distal extreme. The morphology of the most anterior digit most closely resembled digit 1, therefore we propose a loss of an intermediate digit(s). In control embryos, the pelvis anchors the hindlimb to the spine (Fig. 1A,B,E). Strikingly, in mutant embryos, the most proximal elements, the femur and pelvis, were either absent or severely hypoplastic (Fig. 1C,D,F). The most proximal elements that did form (tibia) lacked any connection with the main body axis (Fig. 1C,D,F).

### Dual inputs from *Tbx4* and *Pitx1* are required for hindlimb initiation

At E10.5, *Tbx4^lox/lox^;RetRVCre* hindlimbs buds were smaller than those of controls and expression of *Fgf10* was reduced, although detectable (Fig. S2A,B) demonstrating that, in the absence of *Tbx4*, another factor can partially compensate to regulate *Fgf10* expression. This is in contrast to the forelimb where there is an exclusive requirement for *Tbx5* for initiation of *Fgf10* expression (Rallis et al., 2003). *Pitx1* is a candidate factor to act in addition to *Tbx4* in regulating *Fgf10*. *Pitx1^−/−^* mutant mice form a small hindlimb that has lost some of its characteristic hindlimb morphologies, such as the presence of a patella (Duboc and Logan, 2011a,b; Marcil et al., 2003; Szeto et al., 1999; Fig. 1G). *Pitx1* is known to be partially required for normal levels of *Tbx4* expression (Duboc and Logan, 2011b; Marcil et al., 2003; Szeto et al., 1999), but we found that *Pitx1* expression is unaffected following conditional deletion of *Tbx4* (Fig. S2D,E). We therefore generated *Tbx4*/*Pitx1* compound mutants (*Pitx1^−/−^;Tbx4^lox/lox^;RetRVCre*) and in these compound mutants no hindlimb elements were present (Fig. 1H). Moreover, *Fgf10* expression was not detectable in the hindlimb-forming region at hindlimb budding stages (Fig. S2C). These results demonstrate that *Pitx1* and *Tbx4* have dual inputs that are required for *Fgf10* expression and subsequent hindlimb formation.

### *Isl1* acts in parallel to *Tbx4* and *Pitx1* during hindlimb initiation

**Fig. 2.**
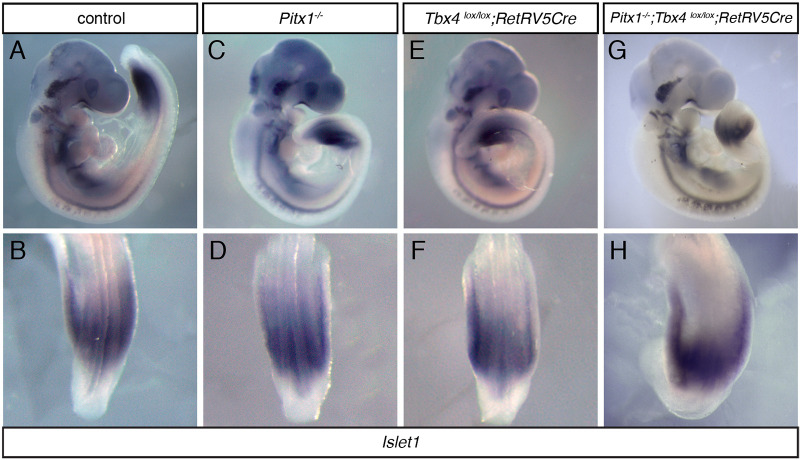
***Tbx4* and *Pitx1* are not required for *Isl1* expression in the hindlimb-forming region.** Whole-mount *in situ* hybridisation for *Isl1* expression in control (A,B; *n*=11), *Pitx1^−/−^* mutant (C,D; *n*=6), *Tbx4^lox/lox^;RetRV5Cre* conditional mutant (E,F; *n*=8) and *Pitx1^−/−^;Tbx4^lox/lox^;RetRV5Cre* compound mutant (G,H) embryos (*n*=2). Upper panels are lateral views of E9.75 embryos. Lower panels are dorsal views of the caudal end of embryos encompassing the hindlimb-forming region.

**Fig. 3.**
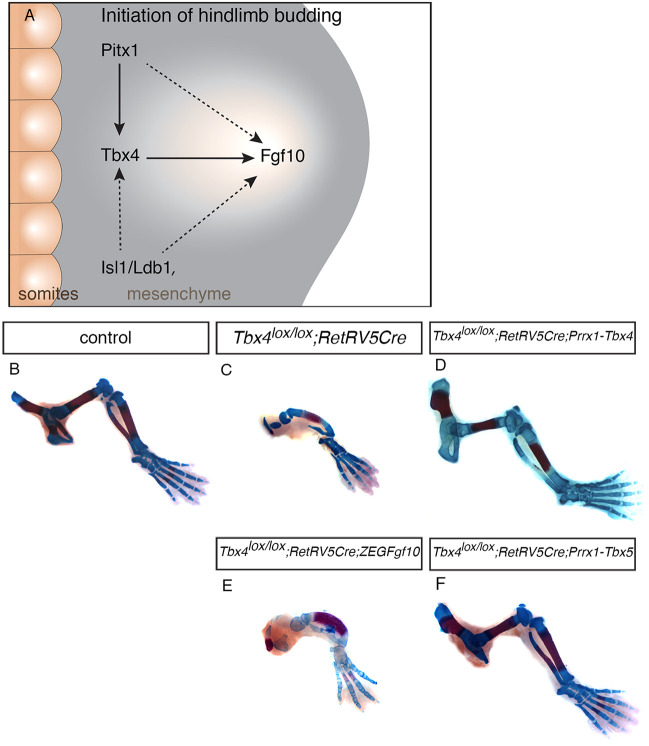
***Tbx5* but not *Fgf10* can rescue hindlimb development in the absence of *Tbx4*.** (A) Schematic of the gene regulatory network operating during the initiation of hindlimb outgrowth. Dashed arrows indicate proposed indirect interactions and solid arrows indicate potential direct interactions. (B-F) Alcian Blue/Alizarin Red skeletal preparations of control (B; *n*=7), *Tbx4^lox/lox^;RetRV5Cre* (C; *n*=8), *Tbx4^lox/lox^;RetRV5Cre;Prrx1-Tbx4* (D; *n*=3), *Tbx4^lox/lox^;RetRV5Cre;Z/EGFgf10* (E; *n*=5) and *Tbx4^lox/lox^;RetRV5Cre;Prrx1-Tbx5* (F; *n*=6) E17.5 hindlimbs.

A third factor that has been implicated in the initiation of hindlimb outgrowth specifically is the LIM homeodomain transcription factor *Isl1* (Itou et al., 2012; Kawakami et al., 2011; Narkis et al., 2012). *Isl1* and its obligatory co-regulators *Ldb1* and *Ldb2* are essential for hindlimb formation through their regulation of *Fgf10* but they are not required for *Tbx4* or *Pitx1* expression (Kawakami et al., 2011; Narkis et al., 2012). *Isl1* expression was unaffected in either the *Pitx1* mutant (*Pitx1^−/−^*), *Tbx4* conditional mutant (*Tbx4^lox/lox^;RetRVCre*) or *Pitx1/Tbx4* compound mutant (*Pitx1^−/−^;Tbx4^lox/lox^;RetRVCre*) (Fig. 2) at hindlimb initiation stage (E10.0), demonstrating that *Tbx4* and *Pitx1* are not required for *Isl1* expression. Therefore, as *Isl1*, *Ldb1* and *Ldb2* do not act upstream or downstream of *Tbx4* and *Pitx1*, we favour a model in which Isl1/Ldb1/2 act as obligate co-factors with Tbx4 and Pitx1, functioning in parallel to regulate *Fgf10* expression (Fig. 3A).

### *Tbx5* but not *Fgf10* can rescue hindlimb proximal defects in the absence of *Tbx4*

*Fgf10* is essential for limb bud formation and it is a target of *Tbx4/5*. To determine whether direct regulation of *Fgf10* expression is the primary function of *Tbx4/5* during the initial phase of limb bud formation, we compared the abilities of *Tbx4* and *Tbx5* and their downstream target, *Fgf10*, to rescue hindlimb formation in the *Tbx4* conditional mutant using a gene deletion/gene replacement strategy. As a control experiment, we crossed transgenic lines expressing either *Tbx4* or *Tbx5* under control of the *Prrx1* regulatory element (*Prrx1-Tbx4* and *Prrx1-Tbx5*; Duboc and Logan, 2011b; Minguillon et al., 2005) into the background of the *Tbx4* conditional knockout (*Tbx4^lox/lox^;RetRVCre;Prrx1-Tbx4* and *Tbx4^lox/lox^;RetRVCre;Prrx1-Tbx5*). Compared with control hindlimb (Fig. 3B) and *Tbx4* conditional knockout hindlimb (Fig. 3C), both *Tbx4* and *Tbx5* transgenes were able to rescue hindlimb development in the *Tbx4* conditional knockout background (Fig. 3D,F). Consistent with our previous observations (Duboc and Logan, 2011b; Minguillon et al., 2005, 2009), the *Tbx5*-rescued limb retained all hindlimb characteristics, indicating that *Tbx5* and *Tbx4* can act equivalently to regulate limb outgrowth and have no role in determining forelimb or hindlimb morphologies in mouse. Using the same strategy with a cre-inducible *Fgf10* transgenic line (*Z/EGFgf10*; see Materials and Methods; Sulaiman et al., 2016), we observed no discernible rescue of the *Tbx4* conditional knockout phenotype (Fig. 3E). This is despite the fact that the same *Fgf10*-inducible line and cre transgenic were able to fully rescue hindlimb formation in the *Fgf10* mutant (*Fgf10^−/−^;RetRvCre;Z/EGFgf10*) (Fig. S3). These results demonstrate that the level of FGF signalling in the hindlimb is not established solely through the direct regulation of Fgf10 ligand by *Tbx4* and that other *Tbx4* targets have crucial roles in establishing FGF signalling levels sufficient for normal limb outgrowth. In the forelimb, *Tbx5* acts in a feed-forward loop to regulate both Fgf10 ligand and mesenchymal expression of an FGF receptor that is required to establish the positive-feedback loop of FGF signalling (Harvey and Logan, 2006). Our results are consistent with an equivalent relationship existing in the hindlimb (Fig. 3A).

### Hindlimb proximal defects in the *Tbx4* mutant cannot be explained by defects in proximal-distal specification, absence of *Fgf8* expression or cell death

**Fig. 4.**
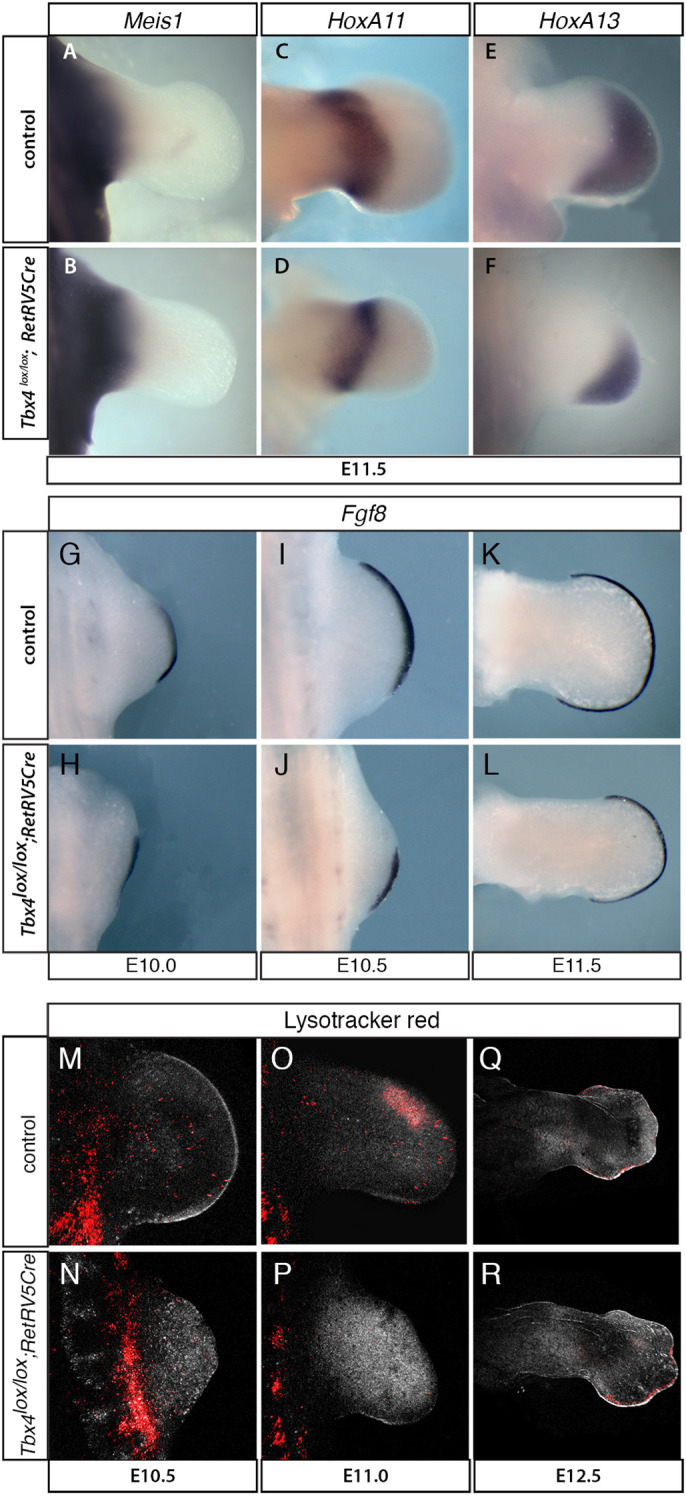
**Disruption of proximal-distal patterning, *Fgf8* expression in the AER and elevated levels of cell death are not observed in the *Tbx4* mutant hindlimbs.** (A-F) Whole-mount *in situ* hybridisation of E11.5 wild-type control (A,C,E) and *Tbx4^lox/lox^;RetRV5Cre* mutant (B,D,F) hindlimbs. The proximal segment (stylopod) marker *Meis1* (A; *n*=3) is expressed in the mutant (B; *n*=3), the medial segment (zeugopod) marker *Hoxa11* (C; *n*=5) is expressed in the *Tbx4* mutant (D; *n*=2) and the distal segment (autopod) marker *Hoxa13* (E; *n*=3) is expressed in the *Tbx4* mutant (F; *n*=6). (G-L) Whole-mount *in situ* hybridisation for *Fgf8* on control hindlimbs at E10 (G; *n*=3), E10.5 (I; *n*=3) and E11.5 (K; *n*=3) and *Tbx4^lox/lox^;RetRV5Cre* mutant hindlimbs at E10 (H; *n*=2), E10.5 (J; *n*=3) and E11.5 (L; *n*=2). (M-R) Whole-mount LysoTracker Red staining for apoptotic cells. Confocal *z*-stacks images of control (M,O,Q) and *Tbx4* mutant (N,P,R) hindlimbs.

The limb skeleton is divided into three anatomical segments from proximal to distal: the stylopod (humerus/femur), zeugopod (radius/tibia and ulna/fibula) and autopod (wrist/ankle and digits) (Fig. 1A) and the genes *Meis1/2*, *Hoxa11* and *Hoxa13*, respectively, are markers of these territories (Galloway et al., 2009; Roselló-Diez et al., 2011; Fig. 4A,C,E). Although the *Tbx4* mutant hindlimbs are smaller, three distinct stylopod, zeugopod and autopod domains could be clearly distinguished with these three markers at E10.5 (Fig. S4A-F) and E11.5 (Fig. 4B,D,F), demonstrating that proximal-distal specification can occur in the absence of *Tbx4* and that proximally biased defects generated in these mutants cannot be explained by failure to establish proximal-distal pattern.

The short stature homeobox gene *Shox2* is expressed in the proximal limb bud and deletion of *Shox2* causes a failure of stylopodal element (e.g. femur in the hindlimb) formation. The absence of femur shares phenotypic similarities with the defects observed in the *Tbx4* mutants we report here, although girdle elements, which are absent in the *Tbx4* mutant, are spared in the *Shox2* null (Cobb et al., 2006; Yu et al., 2007). We observed downregulation of *Shox2* in the *Tbx4* mutant at E10.5 (Fig. S4G,H) consistent with *Shox2* being a target of *Tbx4* (Glaser et al., 2014) and indicating that this may contribute to the absence of femur in the *Tbx4* mutant. Interestingly, however, by E11.5 the proximal domain of *Shox2* was established in the *Tbx4* mutant (Fig. S4I,J); therefore, although *Tbx4* is required to establish the initial domain of *Shox2* at the correct time, *Tbx4* is not essential to establish and maintain *Shox2* expression at later time points.

Conditional deletion of *Fgf8* in the apical ectodermal ridge (AER) generates proximally biased hindlimb skeletal abnormalities. A model to explain these defects proposes that in the absence of FGF signalling in the AER a smaller limb bud emerges but precursors of the proximal segment are more severely affected owing to increased apoptosis in the proximal region (Lewandoski et al., 2000; Mariani et al., 2008; Sun et al., 2002). We compared expression of *Fgf8*, the earliest and predominant FGF expressed in the AER (Mariani et al., 2008), in control hindlimb buds and after *Tbx4* conditional deletion (Fig. 4G-L). Expression of *Fgf8* was initiated at the same stage (E10) in the control and *Tbx4* mutant embryos (Fig. 4G,H); however, the *Fgf8* expression domain was restricted to the posterior AER in the mutant. The posterior restriction in the mutant hindlimb was still evident at E10.5, but by E11.5 the domain of *Fgf8* had extended to encapsulate the distal extreme of the *Tbx4* mutant hindlimb, which is narrower than the control hindlimb. Therefore, an *Fgf8*-expressing AER forms in the *Tbx4* mutant hindlimb despite being initially posteriorly restricted.

To determine whether the alteration in the *Fgf8* expression domain can lead to regionally restricted cell death, as has been suggested previously, we examined cell death directly using whole-mount LysoTracker Red staining and compared control and conditional mutant hindlimbs over a time course from E10.5 to E12.5 (Fig. 4M-R). The number of cells undergoing cell death detected by LysoTracker Red were qualitatively comparable between control and *Tbx4* mutant hindlimbs. Elevated levels of LysoTracker Red staining, as an indicator of cell death, which could account for the absence of proximal structures, were not observed in the proximal regions of mutant limb buds.

Taken together, these results show that neither failure of specification of the proximal segment nor loss of AER FGF expression and resulting increased cell death can explain the proximal bias of the skeletal phenotype. The initial posterior restriction of *Fgf8* expression in the AER seen in the hindlimb bud at E10-E10.5 is, in essence, equivalent to removing the anterior AER. In the chick, following removal of the anterior AER digit 1 fails to develop and frequently the radius is absent but posterior and more proximal structures are unaffected (Todt and Fallon, 1987). Therefore, the initial disruption of anterior AER *Fgf8* expression in the *Tbx4* mutant hindlimbs could account for the absence digits observed but does not explain the proximal defects.

### Sox9-positive chondroprogenitors are present in the proximal *Tbx4* mutant hindlimb

**Fig. 5.**
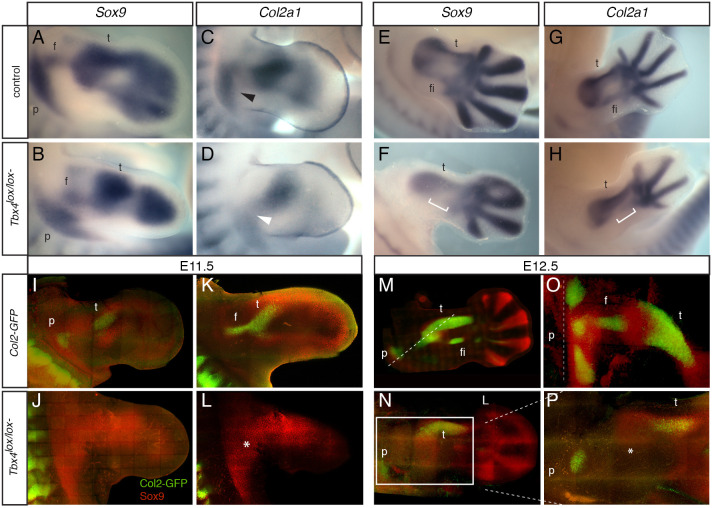
**Sox9-expressing chondroprogenitors are present in proximal *Tbx4* mutant hindlimbs but fail to differentiate into chondrocytes.** (A-H) Whole-mount *in situ* hybridisation showing *Sox9* expression in control (A; *n*=9) and *Tbx4^lox/lox^;RetRV5Cre* (B; *n*=5) hindlimbs at E11.5. *Col2a1* expression in control (C; *n*=4) and *Tbx4^lox/lox^;RetRV5Cre* (D; *n*=2) hindlimbs. *Sox9* expression in control (E; *n*=5) and *Tbx4^lox/lox^;RetRV5Cre* (F; *n*=3) hindlimbs at E12.5. *Col2a1* expression in control (G; *n*=6) and *Tbx4^lox/lox^;RetRV5Cre* (H; *n*=3) hindlimbs. *Col2a1* expression in the pelvic region of the control hindlimb (black arrowhead in C) is reduced or absent in the mutant hindlimb (white arrowhead in D). f, femur; fi, fibia; p, pelvis; t, tibia. Brackets indicate location of absent fibula condensation. (I-P) Whole-mount immunostaining for the chondroprogenitor marker Sox9 (red) in the background of the *Col2-GFP* transgenic mouse reporter, which labels chondrocytes (green) in the forming cartilage. I, J, M, N are 3D renderings of confocal *z*-stacks of images. K, L, O, P are images of a single *z*-plane in the stack. (I,K) Control-*Col2-GFP* hindlimb at E11.5, with condensation of the forming pelvis (p), femur (f) and tibia (t) annotated. These condensations are absent from the *Tbx4^lox/lox^;RetRV5Cre;Col2*-GFP hindlimbs (J,L). The asterisk in L indicates the proximal pool of chondroprogenitors. (M,O) Control *Col2-*GFP hindlimb at E12.5 with pelvis, tibia and fibula (fi) condensations indicated. (N,P) *Tbx4^lox/lox^;RetRV5Cre;Col2-*GFP hindlimbs. Dashed line in M indicates the plane of *z*-section through the limb. Boxed area in N is shown at higher magnification in P. Asterisk in P indicates the absence of femur condensation.

One explanation for the absence of proximal elements is that the pool of chondroprogenitors that gives rise to these structures fails at a step of chondrogenesis. To test this hypothesis, we examined the expression of *Sox9*, a marker of committed chondroprogenitors, and collagen 2a1 (*Col2a1*), an early marker of differentiating chondrocytes (Fig. 5A-H). *Sox9*-expressing cells were detected throughout the proximal-distal extent of *Tbx4* mutant limbs and, significantly, were present in the most proximal regions (Fig. 5B) where in the equivalent region of the control the precursors of the pelvis and femur can be detected (Fig. 5A). At the same stage, in the control, these proximal cells were beginning to express *Col2a1*; however, in the *Tbx4* mutant *Col2a1* was barely detectable (compare Fig. 5C,D). In contrast, in more distal regions at E12.5 the absence of skeletal elements (e.g. fibula and some digits) was associated with a failure to express both *Sox9* and, subsequently, *Col2a1*, indicating that different mechanisms operate in proximal and distal regions leading to loss of structures in the mutant (Fig. 5E-H).

To examine the proximally localised block in chondrogenesis more closely, we analysed Sox9 protein levels by immunostaining in the background of a *Col2*-GFP reporter line. Sox9-positive chondroprogenitor cells were present in both proximal and more distal parts of the mutant hindlimb at E11.5 (Fig. 5J,L), but initial condensations of Col2-positive chondrocytes that eventually form the pelvis, femur and tibia, and could be seen in control (Fig. 5I,K), were absent in the mutant (Fig. 5J,L). By E12.5, rods of Col2-positive cells were clearly visible in the forming pelvis, femur and tibia elements of control hindlimbs (Fig. 5M,O), whereas in the mutant only the tibia and a smaller number of dispersed cells in the pelvic region were present (Fig. 5N,P). These results indicate that in the *Tbx4* mutant, Sox9-positive chondroprogenitors are present in the proximal limb bud but these cells fail to take the next steps in the chondrogenic programme and do not express Col2a1.

### In the absence of *Tbx4*, chondroprogenitors located in the proximal part of the limb bud fail to differentiate into chondrocytes

**Fig. 6.**
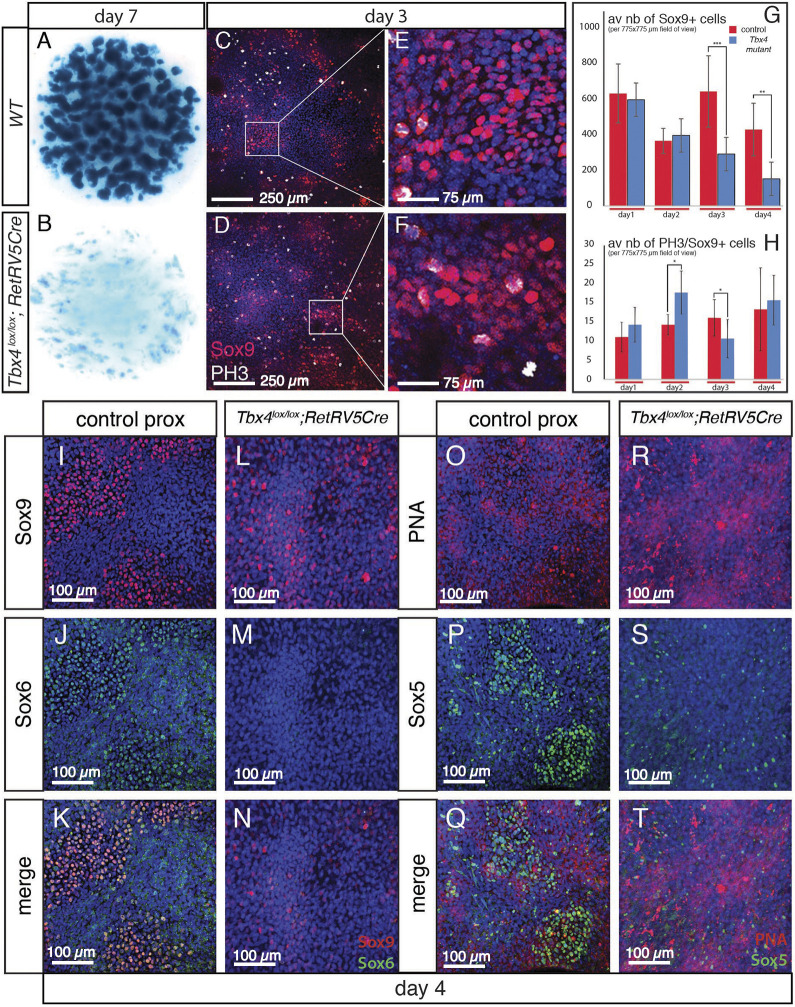
***Tbx4^lox/lox^;RetRV5Cre* chondroprogenitors fail to differentiate into chondrocytes.** (A,B) Alcian Blue staining of cartilage nodules in day 7 micromass control proximal (A; *n*=14) and *Tbx4^lox/lox^;RetRV5Cre* (B; *n*=11) cultures. (C-F) Sox9 immunostaining (red) and DAPI nuclear staining (blue) of day 3 proximal limb control (C,E) and *Tbx4^lox/lox^;RetRV5Cre* (D,F) cultures. (E) Higher magnification of a forming nodule (boxed in C) showing rounded Sox9-positive cells. (F) Higher magnification of *Tbx4^lox/lox^;RetRVCre5* culture (boxed in D) showing Sox9-positive aggregate of cells that do not have a rounded morphology. (G) Histogram showing number of Sox9-expressing cells (mean±s.e.m.) in control proximal micromass (red) cultures and *Tbx4^lox/lox^;RetRV5Cre* proximal culture (blue) per 775×775 µm. These numbers are equivalent at day 1 and progressively decrease as compaction proceeds around day 3 of culture. (H) Histogram showing number of proliferating (PH3-expressing) cells (mean±s.e.m.) among Sox9-expressing cells from day 1 to day 4 of culture. ***P<0.001, **P<0.01, *P<0.05 (unpaired, two-tailed Student's *t*-test; *n*=5). (I-T) Double immunostaining of proximal limb mesenchymal cell cultures (4 days) showing expression of Sox9 (I,K,L,N) and Sox6 (J,K,M,N) in wild-type proximal control (I-K; *n*=3) and *Tbx4^lox/lox^;RetRV5Cre* proximal (L-N; *n*=3) cultures. Nuclear staining (DAPI) is shown in blue. (O-T) Peanut agglutinin (PNA) staining, as a marker of chondroprogenitor cells undergoing condensation (O,Q,R,T; *n*=3) and Sox5 immunostaining (P,Q,S,T; *n*=3) in wild-type proximal control (O-Q; *n*=5) and *Tbx4^lox/lox^;RetRV5Cre* proximal (R-T; *n*=3) cultures. Nuclear staining (DAPI) is shown in blue.

To investigate which step of the chondrocyte differentiation process is affected in the absence of *Tbx4*, we used the micromass cell culture technique (see Materials and Methods). This system allows limb bud cells to be studied in isolation from the influence of AER signals and the forming vasculature. If cultured *in vitro* under the correct conditions, limb bud cells are able to differentiate to form cartilage nodules that can be stained with Alcian Blue (Fig. 6A). In *Tbx4* mutant micromass cultures made from the proximal portion of the limb bud, there was an almost complete absence of staining (Fig. 6B), indicating a failure to form cartilage. In contrast, equivalent cultures produced from the distal portion of *Tbx4* mutant limbs were able to form cartilage nodules, although not as effectively as control samples (Fig. S5A,B). This demonstrates that proximal cells in the *Tbx4* mutant have a more severe block in their ability to undergo chondrogenesis than distal cells.

We next examined the chondrogenic differentiation programme in *Tbx4* mutants to determine the point at which it is disrupted. In the first steps of chondrogenic differentiation, the Sox9-expressing, prechondrogenic mesenchymal cells aggregate and subsequently condense to form compact cell masses that go on to form cartilage (Fig. 6C-F). *In vitro* differentiation of chondroprogenitors has been shown to depend on the density of progenitors present in culture (Ahrens et al., 1977). To assess whether the phenotype observed is derived from a lower number of progenitors in the harvested limb cells, we quantified the average number of Sox9-expressing cells in culture from day 1 to day 4 of culture (Fig. 6G,H). On day 1 and 2, we observed no statistical difference in the number of Sox9-positive progenitors between controls and the mutant condition, suggesting that a difference in the initial cell density of progenitors is not the primary cause of the phenotype observed. However, interestingly, the number of Sox9-positive cells decreased from day 3 of culture, concomitantly with the onset of the compaction process. Defects of cell proliferation cannot account for the decreased number of Sox9-positive cells observed in the absence of *Tbx4* expression as comparable numbers of phospho-histone H3 (PH3)-positive cells were present in both Sox9-positive mutant and control populations (Fig. 6H). Quantification of caspase 3 immunostaining showed that there is no increase of apoptosis in the mutant cell cultures (Fig. S5C-E), suggesting that the reduction in the number of Sox9-positive cells is due to chondroprogenitor cells failing to maintain Sox9 expression rather than these cells being lost via cell death.

Two additional Sox factors, *Sox5* and *Sox6*, are co-expressed with *Sox9* during chondrogenic differentiation (Ikeda et al., 2005). These transcription factors act after mesenchymal condensation has occurred and cooperate with Sox9 to activate the *Col2a1* enhancer and allow chondrocyte differentiation (Smits et al., 2001). At day 4 of micromass culture, Sox6/Sox9 co-expressing cells could be detected (Fig. 6I-K) but in cultures of *Tbx4* mutant cells, Sox9-expressing cells did not co-express Sox6 (Fig. 6L-N). Similarly, using peanut agglutinin (PNA) to detect condensing cartilage nodules, Sox5 was observed in control cultures (Fig. 6O-Q) but was not detected in *Tbx4* mutant cultures even in regions rich for PNA (Fig. 6R-T). Therefore, in *Tbx4* mutants, cartilage formation is disrupted at an early stage.

### Sox9-positive chondroprogenitors fail to undergo compaction in *Tbx4* mutant cultures

**Fig. 7.**
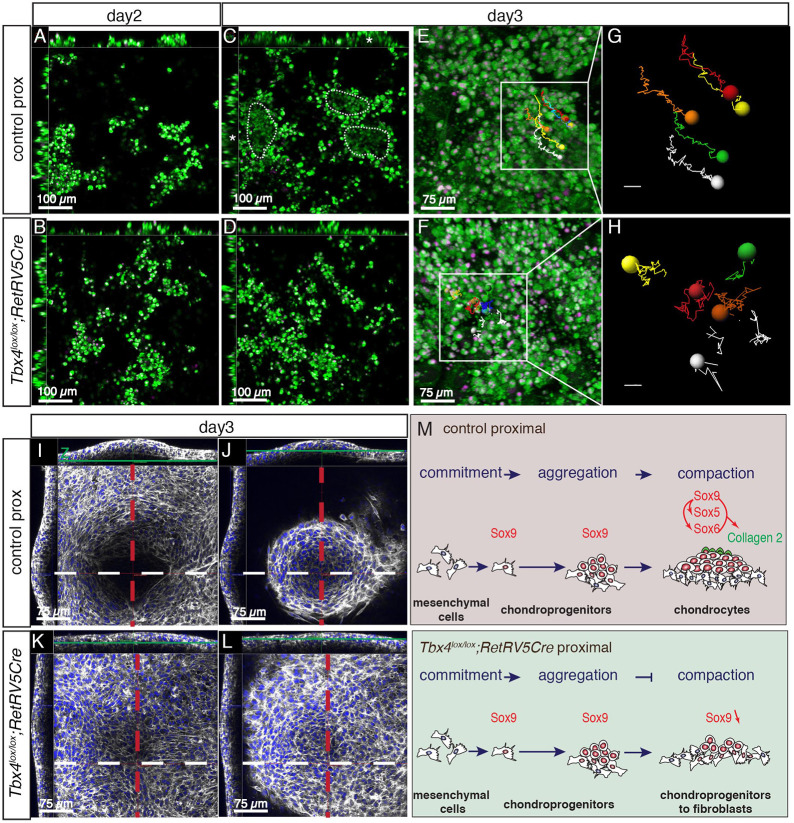
***Tbx4* mutant chondroprogenitors fail to undergo compaction in micromass culture.** (A-F) Confocal images extracted from an 84 h time-lapse analysis of control (A,C,E) and *Tbx4^lox/lox^;RetRV5Cre* (B,D,F) proximal limb bud micromass culture. (A-D) *x*, *y* and *z* views of the same cultures. *z* views in A-D are a single scan through approximately the middle of the stack. *x* and *y* views show the entire *z* stack along lines though the cultures. At day 2 of culture, both control (A) and *Tbx4* mutant (B) cultures form cell aggregates. At day 3, cells at the centre of aggregates (C) lose their contacts with the substrate and lift off the dish while retaining close contacts with one another. A void is created under the forming nodule (asterisks) below the centre of the cell aggregate. This behaviour is coincident with the onset of compaction. Three examples are encircled (dotted lines) in panel C. In *Tbx4* mutant cultures (D), cell aggregates maintain connections with the dish, cells fail to lift off the dish and the compaction process is not evident. (E,F) Extended focus images of *z*-stacks showing the area culture were cells were tracked. (G,H) Tracks of cells over 7 h 15 m in wild-type (G) and *Tbx4* mutant (H) cultures. (I-L) Day 3 cultures stained for nuclei (DAPI, blue) and F-actin (phalloidin, white). Each panel shows confocal *x*, *y* and *z* views of focal planes through levels at the base of the culture (I,K) and top of the culture (J,L). The control cultures have distinctive features. A void forms under the forming nodule (I). At both the base (I) and top (J) of the culture, cells of the forming nodule are arranged in a circular pattern with tightly packed, rounded cells at the centre that have lost their contacts with the dish surface. In *Tbx4* mutant cultures (K,L), cells are arranged randomly, have retained their contacts to the dish and do not lift off the dish. (M) Summary of the chondrocyte differentiation process in control and in *Tbx4^lox/lox^;RetRV5Cre* micromass cultures. In control cultures aggregates of Sox9-positive cells expand and undergo compaction before undergoing chondrocyte differentiation and expressing collagen 2. In *Tbx4* mutant cultures, aggregates of Sox9-positive cells fail to undergo compaction, Sox9 expression is lost and cells do not undergo chondrocyte differentiation.

To examine the cellular behaviour of chondrocytes at the earliest stages of chondrocyte condensation when phenotypes have been observed, we produced time-lapse movies of proximal limb micromass cultures from day 1 to early day 4 (84 h), a time window encompassing the onset of the differentiation process. Cultures were stained with a cytoplasmic dye and a low concentration of Hoechst to follow nuclei (see Materials and Methods). In the control proximal cell culture at day 1, cells had adhered to the plate and had a classic fibroblast-like morphology (Movies 1 and 2). At day 2, subsets of cells changed from a flattened morphology, formed aggregates with neighbouring cells and appeared to loosen their contacts to the substrate. This process of cell aggregation, which is thought to be the first step of the condensation process (Barna and Niswander, 2007) and precedes the onset of *Sox9* expression, was observed in both control and *Tbx4* mutant cultures (Fig. 7A-D; Movies 1 and 2). By day 3 in control cultures, cells at the core of aggregates lost their contacts with the dish and lifted up, creating a void at the base of the forming nodule (Fig. 7C, asterisks; Movie 1). In *Tbx4* mutant cultures at day 3, cells within aggregates maintained their contacts with the dish and compaction of the chondroprogenitors failed to occur (Fig. 7D). By day 3 in control cultures, cells in aggregates stopped exchanging neighbours, as shown by their almost parallel motion revealed by manual tracking of cells in aggregates of wild-type cultures (Fig. 7E,G). In *Tbx4* mutant cultures at day 3, cells within aggregates maintained their contacts with the dish and continued to change positions in the aggregate, suggesting that they are unable to maintain stable cell-cell adhesion (Fig. 5D,F,H).

To analyse further the distinct topography and cellular organisation of micromass cultures, we generated confocal *z* stack scans of cultures stained with phalloidin (for F-actin) and DAPI (for nuclei). *x*- and *y*-axis views (Fig. 7I,J) illustrate that the nodule is a raised area of cells within the culture. This is the result of cells at the core of the nodule losing their contacts with the surface of the dish producing an acellular void beneath the nodule. In normal micromass culture, the appearance of an acellular void corresponds to the accumulation of extracellular matrix. The absence of acellular voids in the *Tbx4* mutant micromass cultures could therefore indicate a failure of, or reduction in, the production of extracellular matrix. At both the base and top of the culture (Fig. 7I and 7J, respectively), cells of the forming nodule were arranged in a circular pattern with tightly packed, rounded cells at the centre. In contrast, *Tbx4* mutant cultures remained flat. Cells retained their contacts with the substrate and were arranged randomly (Fig. 7K,L). Thus, *Tbx4* mutant cells exhibit defects in the very earliest stages of chondrocyte differentiation, which leads to the failure of cartilage elements to form properly in this region and ultimately results in proximally biased defects in the hindlimb.

## DISCUSSION

### *Fgf10* expression is regulated by different mechanisms in the forelimbs and hindlimbs

Establishment and maintenance of a positive-feedback loop of FGF signalling between cells of the limb mesenchyme and AER is essential for limb bud outgrowth and elaboration of the proximal-distal sequence of skeletal elements. This is triggered by expression of Fgf10 ligand in nascent forelimbs and hindlimbs. Deletion of *Fgf10* causes a failure of limb bud formation and absence of almost the entire limb skeleton with the forelimb and hindlimbs being affected similarly. Our results demonstrate that, although *Fgf10* has equivalent roles in both forelimb and hindlimbs, there are differences in how *Fgf10* expression is regulated in each type of limb. In the forelimb, *Tbx5* is exclusively required for *Fgf10* expression. In the *Tbx5* mutant, *Fgf10* expression is not initiated and, consequently, all forelimb elements fail to form (Rallis et al., 2003). There is not the same requirement for a Tbx input in the hindlimb as our results demonstrate that in the absence of *Tbx4*, low levels of *Fgf10* expression are established and ultimately distal hindlimb elements are produced whereas more proximal elements are missing. Furthermore, we show that, following deletion of both *Tbx4* and *Pitx1*, *Fgf10* is not expressed and all hindlimb elements fail to form. We propose a model in which *Pitx1* has dual, separable inputs in the regulation of *Fgf10* (Fig. 3A). *Pitx1* positively regulates *Tbx4*, which in turn directly regulates *Fgf10*. *Pitx1* also has a *Tbx4*-independent input into the regulation of *Fgf10* that can establish hypomorphic levels of Fgf10 in the *Tbx4* mutant. Although *Isl1* and *Ldb1/2* are required for *Fgf10* expression and are still expressed in the *Tbx4*/*Pitx1* double mutant, our results demonstrate that they are not sufficient to rescue *Fgf10* expression; we therefore favour a model in which these factors act as obligate co-factors with Tbx4 and Pitx1 to regulate *Fgf10*.

In our *Tbx4* gene deletion/gene replacement assay, both *Tbx4* and *Tbx5* can rescue hindlimb formation equally well and a morphologically indistinguishable hindlimb is formed in each case, consistent with our previous observations that these genes have no role in determining forelimb or hindlimb morphologies in mouse (Duboc and Logan, 2011b; Minguillon et al., 2005, 2009). Recently, in an avian model, the pigeon, cis regulatory alleles mapping to *Tbx5* (and *Pitx1*) have been mapped to loci associated with feathered feet that are believed to represent partial transformations from hindlimb to forelimb identity (Boer et al., 2019; Domyan et al., 2016). Whether this represents a difference between avians and mammals in how differences between forelimbs and hindlimb morphologies are established remains to be clarified. Significantly, the immediate downstream target of *Tbx4/5*, *Fgf10*, did not produce any detectable rescue of hindlimb formation indicating that Tbx targets other than *Fgf10* are required to establish the FGF positive-feedback loop. Previously, we have demonstrated that in the forelimb *Tbx5* acts in a feed-forward loop with *Sall4* to establish FGF signalling (Harvey and Logan, 2006). Our results are consistent with *Tbx4* acting in an equivalent feed-forward mechanism in the hindlimb.

In the rescue assays that we describe here and have reported previously (Duboc and Logan, 2011b; Minguillon et al., 2005, 2009), *Tbx4* and *Tbx5* are equally efficient in rescuing either forelimb or hindlimb formation. Therefore, it remains unclear if there is any functional advantage in acquiring the additional inputs of *Pitx1* and *Isl1*/*Ldb*, in addition to *Tbx4*, for the regulation of *Fgf10* in the hindlimb. In the context of limb evolution, these additional regulatory inputs provide alternative targets for modulation, such as that described in stickleback pelvic fin reduction (Chan et al., 2010; Infante et al., 2013; Jones et al., 2012; Shapiro et al., 2004), and they enable reduction in pelvic (hindlimb) appendages without the pectoral appendages (forelimb) being affected.

### A mechanism for proximal, skeletal limb defects

In the absence of *Tbx4* expression, proximal chondroprogenitors expressing Sox9 do not form aggregates *in vitro* but subsequently fail to undergo compaction and further steps of chondrogenesis (Fig. 7M). These cells then progressively decrease Sox9 expression levels. Tbx4 could be acting by regulating signalling pathways known to contribute to cartilage formation in the limb. In chick micromass culture, inhibition of BMP signalling results in failure of chondroprogenitor compaction, at a stage similar to that affected in the *Tbx4* mutant we report here. Consistent with this, in mouse, BmpR1a and -b are required for the expression of *Sox9*, *Sox6* and *Sox5*. In contrast, in the *Tbx4* mutant we show that *Sox9* expression is induced but not maintained, suggesting that *Tbx4* could act downstream of BmpR1 activity. The canonical Wnt pathway can affect chondrogenic differentiation. Following ectopic Wnt expression in chick micromass culture, chondrocytes undergo compaction but their differentiation into chondroblasts is blocked (Day et al., 2005; Rudnicki and Brown, 1997). Furthermore, conditional deletion of β-catenin in the mouse results in increased Sox9 expression and an increase in the number of chondrocytes, at the expense of osteoblasts (Day et al., 2005). Both of these processes occur after the compaction of chondroprogenitors, the step we see affected in the *Tbx4* mutant, therefore ruling out the Wnt pathway as a mediator of this observed defect. Both *Tbx4* and *Tbx5* are known to contribute to the initiation of *Fgf10* expression in the limb mesenchyme (Hasson et al., 2007; Minguillon et al., 2005; Rallis et al., 2003). FGFs are required for the viability of the chondrogenic precursor pool that gives rise to the cartilaginous templates. Our results show no statistically significant increase of cell death both *in vivo* and *in vitro* when *Tbx4* is deleted, suggesting *Tbx4* activity on chondrogenic precursors is independent of FGF signals. In agreement with this observation, mice lacking *Fgf10* do not form limb buds but do form rudimental girdle structures (scapula and pelvis) (Sekine et al., 1999; Fig. S1). Girdle elements are absent from both *Tbx5* and *Tbx4* mutant mice, arguing that *Tbx4/*5 but not *Fgf10* activity is required for the formation of these most proximal elements.

Phocomelia is a congenital limb malformation in which the proximal portion of the limb (humerus/femur and girdle) is absent or poorly developed leaving the more distal structures, which are less affected, attached directly to the trunk. Phocomelia can be caused by either genetic mutations or environmental insults. Phocomelia cases also present sporadically and the causes of these cases are often never determined. At least 25 human syndromes can present with phocomelia. Eight of these conditions have a known affected gene association, for example Holt–Oram syndrome (OMIM: 142900), caused by mutations at the *TBX5* gene locus, can present with upper limb phocomelia (Bermejo-Sánchez et al., 2011). The abnormalities produced following conditional deletion of both *Tbx4* alleles in the hindlimb are similar to the defects found in human lower limb phocomelia (Bermejo-Sánchez et al., 2011). The most severely affected skeletal elements are also the same as those affected in human ischiocoxopodopatellar syndrome (OMIM: 147891), an autosomal dominant disorder caused by mutation in the *TBX4* gene. The main clinical features of this syndrome include anomalies of the pelvis and femur, aplastic or hypoplastic patella and anomalies of the feet that are believed to originate from *TBX4* haploinsufficiency (Bongers et al., 2004; Haarman et al., 2019). In addition, *TBX4* homozygous null mutations have been reported to lead to posterior amelia with pelvic hypoplasia (Kariminejad et al., 2019). Hindlimb developmental abnormalities, including clubfoot and tibial hemimelia, have also been associated with deletions or missense mutations in *PITX1* (Alvarado et al., 2011; Gurnett et al., 2008; Klopocki et al., 2012; Morel et al., 2020; Rosenfeld et al., 2011). Our observations in the mouse model provide explanations for the defects, particularly the pelvic and femur involvement, observed in these different human conditions.

Studies in animal models have provided clues to which steps of the limb development programme are disrupted leading to phocomelia. Following X-ray irradiation of chick limb bud, a proximally truncated limb forms (Wolpert et al., 1979). A more recent re-examination of this phenotype suggests that it is caused by selective depletion of proximal chondrocytes that undergo cell death following their exposure in a time window when prechodrogenic progenitors commit to differentiation (Galloway et al., 2009). We demonstrate that in the *Tbx4* mutant levels of cell death do not increase in the proximal compartment during stages preceding or following the events of cartilage condensation and, therefore, cell death cannot explain the absence of proximal elements.

Significantly, we demonstrate that defects in proximal skeletal elements can result from a failure of proximal Sox9-positive chondroprogenitors to differentiate into chondrocytes (Fig. 7M) rather than by increased levels of cell death of limb proximal mesenchymal cells or a disruption in proximal-distal patterning. In a *Gli3*;*Plzf* mouse mutant, which displays a similar loss of proximal skeletal structures, *Sox9* expression is lost and cell death is restricted to the proximal hindlimb. *Gli3* and *Plzf* (*Zbtb16*) are suggested to establish the spatial and temporal distribution of chondrogenic progenitors in the proximal hindlimb in early limb development (Barna et al., 2005). Moreover, *Plzf* has been identified as an upstream regulator of *Sox9* (Djouad et al., 2014); hence, this could explain the absence of *Sox9* in the proximal *Gli3;Plzf* mutant hindlimb bud, in contrast to the *Tbx4* conditional mutant we report here. An additional study has identified significant defects in the hindlimb stylopod and zeugopod in a *Sall4;Plzf* double knockout mutants (Chen et al., 2020). More recently, a study identifying thalidomide-dependent interaction mediated through the ubiquitin-ligase cerublon (Crbn) has shown Plzf and Sall4 to be degraded following thalidomide treatment, leading to hypoplasia in chicken limbs (Chen et al., 2020; Yamanaka et al., 2021). Yamanaka et al. rescue the hypoplastic phenotype by overexpressing Plzf, which recovers the expression of *Fgf10* and *Fgf8*. This study concludes that species sensitive to thalidomide produce Crbn-dependent teratogenic phenotypes and the resultant effect cannot be simply explained by a single knockout of *Plzf* or *Sall4* in mouse models.

Current models suggest that proximal-distal positional values are specified early during limb bud formation (Dudley et al., 2002; Mercader et al., 2000; Towers et al., 2012) and that the action of FGFs expressed in the AER serve to expand the number of progenitors in the limb segments so that structures differentiate in a proximal-to-distal sequence as limb bud outgrowth progresses. The two-signal model for proximal-distal limb patterning incorporates a second component and proposes that proximal structures are specified by retinoic acid from the flank. Outgrowth of the limb bud takes cells out of the range of the proximal source of retinoic acid, allowing FGFs from the AER to specify distal structures and maintain cell survival (Roselló-Díez et al., 2014, 2011; Towers et al., 2012). Our results demonstrate that the proximal-distal markers *Meis1*, *Hoxa11* and *Hoxa13* are expressed in the *Tbx4* mutant and, therefore, that the phocomelia that develops is not caused by disruption of proximal-distal patterning and a failure to specify proximal cell fates.

To explain the occurrence of phocomelia in *Fgf8* null mice, it has been proposed that all three segments of the limb bud have reduced proportions owing to the smaller limb bud size but that the proximal domain is reduced even further by elevated levels of proximal cell death due to decreased AER-FGF signalling. Ultimately, because proximal progenitors have less time to expand before condensation occurs, the femur is more severely compromised than other elements (Mariani et al., 2008). As *Tbx4* is required for normal expression of *Fgf10* in the mesenchyme, which in turn is necessary to induce *Fgf8* in the overlying ectoderm, it is conceivable that hypomorphic levels of FGF signalling could contribute to the emergence of phocomelia. *Tbx4* and *Tbx5* are only required for the initial induction of *Fgf10* expression and subsequent establishment of the FGF positive-feedback signalling loop but are dispensable for further limb outgrowth (Hasson et al., 2007; Minguillon et al., 2005; Naiche and Papaioannou, 2007). Deletion of *Tbx4* at later limb bud stages using the *Prx1Cre* line also produces proximally truncated limbs (Naiche and Papaioannou, 2007), suggesting that disrupted FGF signalling might not be the explanation for this phenotype and instead it arises through a block in chondrogenesis as we describe here.

## MATERIALS AND METHODS

### Embryos and mouse lines

Mouse embryos were staged according to Kaufman (1992). Noon on the day a vaginal plug was observed was taken to be E0.5 days of development. The *Tbx4^lox/lox^* (Naiche and Papaioannou, 2007), *Pitx1*^−/−^ (Szeto et al., 1999), *Fgf10^−/−^* (Sekine et al., 1999), *Prrx1*-*Tbx4, Prrx1-Tbx5* (Minguillon et al., 2009), *Col2*-GFP (Cho et al., 2001), Z/EG (Novak et al., 2000) mutants have all been described previously. The *Z/EGFgf10* and *RetRV5Cre* deleter transgenic lines were generated by the Procedural Services Section, NIMR (see Figs S1 and S3).

### Skeletal preparations

The cartilage and bone elements of mouse embryos were stained with Alcian Blue and Alizarin Red, respectively, essentially as previously described (Hogan et al., 1994). The numbers of samples processed were as follows: E17.5 control (*n*=7), *Tbx4^lox/lox^;RetRV5Cre* (*n*=7), *Tbx4*^Δ*/lox*^*;RetRV5Cre* (*n*=3), *Pitx1^−^****^/−^*** (*n*=7), *Pitx1^−^****^/−^****;Tbx4^lox/lox^;RetRV5Cre* (*n*=2), *Tbx4^lox/lox^;RetRV5Cre;Prrx1-Tbx4* (*n*=3), *Tbx4^lox/lox^;RetRV5Cre;Z/EGFgf10* (*n*=5), *Tbx4^lox/lox^;RetRV5Cre;Z/EGFgf10* (*n*=5), *Tbx4^lox/lox^;RetRV5Cre;Prrx1-Tbx5* (*n*=6), *Fgf10^−/^*^−^ (*n*=1), *Fgf10^−/−^;RetRV5Cre;Z/EGFgf10* (*n*=2).

### RNA *in situ* hybridisation

Whole-mount and section *in situ* hybridisation protocol has been described previously (DeLaurier et al., 2006; Riddle et al., 1993). The following probes have been previously published: *Tbx4*, *Pitx1* and *Hoxa13* (DeLaurier et al., 2006; Riddle et al., 1993), *Fgf10* (Bellusci et al., 1997), *Col2a1* (Metsaranta et al., 1991), *Sox9* (Kent et al., 1996), *Fgf8* (Mahmood et al., 1995), *Meis1a* (Capdevila et al., 1999; Mercader et al., 2000). *Isl1* and *Hoxa11* probes were generated from I.M.A.G.E clones (*Ils1*: IRCLp5011A0814D, I.M.A.G.E ID 40130540; *Hoxa11*: IRCLp5011D086D, I.M.A.G.E ID 8734051). Numbers of embryos processed with each probe are given in the respective figure legends.

### Whole-mount immunohistochemistry

Immunohistochemistry was performed as previously described (DeLaurier et al., 2006) and tile *z*-scanned by confocal microscope (MPSP5, Leica) using a 20× magnification water-dipping lens (numerical aperture 1.0; Leica, 507701). The resulting images were analysed using Fiji (ImageJ) and Volocity (6.1.1, Perkin Elmer) software. Prior to confocal imaging, embryos were cleared using Clear^T2^ as described (Kuwajima et al., 2013). Sox9 protein was detected using anti-human SOX9-NL557 (R&D Systems, NL3075R; 1:10).

### LysoTracker Red

Embryos were dissected in HBSS (Hank's balanced salt solution) and placed in 5 µl/ml of LysoTracker Red (Invitrogen, L7528) for 45 min at 37°C. Embryos were then washed three times for 10 min rocking at room temperature then fixed in 4% paraformaldehyde in PBS overnight. Rinsed embryos were then cleared and imaged using a Zeiss LSM5 Pascal confocal microscope.

### Micromass cultures

Micromass cultures were prepared as previously described (Ahrens et al., 1977; Bruce et al., 2010) from samples of pooled limbs. Hindlimbs were harvested from 11.5 days post-coitum *Tbx4^lox/lox^;RetRV5Cre* conditional mutants and wild-type embryos. Hindlimb buds dissected from the flank of the embryo were bisected transversely at the approximate proximal-distal midpoint to generate proximal and distal portions that were processed separately. Limbs and limb portions were dissociated in 1 unit/ml of dispase II (Roche Diagnostics) solution containing 10% fetal bovine serum (FBS)/Puck's saline A buffer for 20 min at 37°C. Digested limbs were dissociated in growth medium [advanced DMEM/F-12 1:1 medium containing 10% FBS-gold, glutaMAX, Penicillin (25 units/ml), Streptavidin (25 μg/ml) antibiotics, Invitrogen], passed through a 40-μm cell strainer to obtain a single-cell suspension, and centrifuged at 800 ***g*** for 10 min. Cells were resuspended in growth medium at a concentration of 2.5×10^5^ cells/ml and spotted in 10 μl droplets on Nunclon Delta surface culture dishes. After cells adhered to culture dishes for 1 h at 37°C in a humidified atmosphere containing 5% CO2, medium was added. Growth medium was replaced every 2 days.

### Staining and immunostaining of micromass cultures

For Alcian Blue staining, micromass cultures [control proximal culture (*n*=14) *Tbx4^lox/lox^;RetRV5Cre* proximal culture (*n*=11), control distal culture (*n*=6), *Tbx4^lox/lox^;RetRV5Cre* distal culture (*n*=6)] were fixed in 4% paraformaldehyde for 30 min then washed briefly in PBS and incubated in 0.1% Alcian Blue GX (Sigma-Aldrich) in 0.1 N HCl, pH 1.0, overnight at room temperature. Cultures were cleared in 70% ethanol before images were captured on a stereomicroscope. For immunostaining, micromass cultures were fixed for 15 min, washed in PBS, blocked in 10% sheep serum in PBS. Antibodies were added overnight in blocking solution. Anti-human SOX9-NL557 antibody (R&D Systems, NL3075R; 1:10), anti-Sox6 antibody (Abcam, ab30455; 1:250), anti-Sox5 antibody (Santa Cruz Biotechnology, sc-17329; 1:200), PNA-FITC (Sigma-Aldrich, L7381; 20 µg/ml final concentration), anti-phosphohistone H3 (Abcam, ab10543; 1:100), anti-activated caspase 3 (Abcam, ab32351; 1:200) and Alexa Fluor 647 phalloidin (Molecular Probes, A22287; 50 µg/ml). Immunostained cultures were *z*-scanned by confocal microscope (MP-SP5, Leica) using a 20× magnification water-dipping lens (numerical aperture 1.0; Leica, 507701). The resulting images were analysed and cell numbers and volumes were obtained using velocity (6.1.1, Perkin Elmer). Multi-channel stacks were processed on Volocity for 3D rendering and, subsequently, volume quantification and cell counting. For each field of view (775×775 µM) (eight fields in total representing two different biological repeats for each condition and culture day), to perform segmentation and automated cell counting, we used the following protocol: velocity measurements: find objects using intensity>exclude objects touching edge of image>exclude objects by size. The overlaps between the population of PH3- and Sox9-expressing cells was then assessed. Statistical analysis (mean, s.e.m. and unpaired two-tailed Student's *t*-test) was performed using Excel (Microsoft).

### Cell labelling and live imaging of micromass cultures

One-day-old micromass cultures were incubated in CellTracker dye (Invitrogen Molecular Probes, CMTPX, C34552) diluted to a final concentration of 2.5 µM in pre-warmed DPBS (Gibco, 14287080) for 30 min at 37°C. Hoechst-33342 (1 µg/ml; Invitrogen) was used to label nuclei by incubating the cells for the last 5 min of the 30 min incubation. Cells were placed in fresh media and incubated for 1 h prior to imaging. Movies are time lapses of *z*-scan stacks (1.5 µm steps, 30 µm thick) imaged every 5 min using an MPSP5 (Leica) confocal microscope with a 20× magnification water-dipping lens (numerical aperture 1.0; Leica, 507701) equipped with cell culture chamber. Wild-type, control cultures and mutant cultures (*Tbx4^lox/lox^;RetRV5Cre*) were imaged concomitantly in the same Petri dish (60 mm Nunclon delta surface). Tracking of cells in movies was performed manually using IMARIS 8.2.0 (Bitplane) for 80 frames (7H15 m) before the onset of cell compaction.

## Supplementary Material

Supplementary information

Reviewer comments
