# Supplementary material for: Tbx4 function during hindlimb development reveals a mechanism that explains the origins of proximal limb defects
Source: Development. 2021 Sep 24;148(19):dev199580. doi: 10.1242/dev.199580 (PMC8497778; doi:10.1242/dev.199580)
Supplement: Supplementary information [file develop-148-199580-s1.pdf]

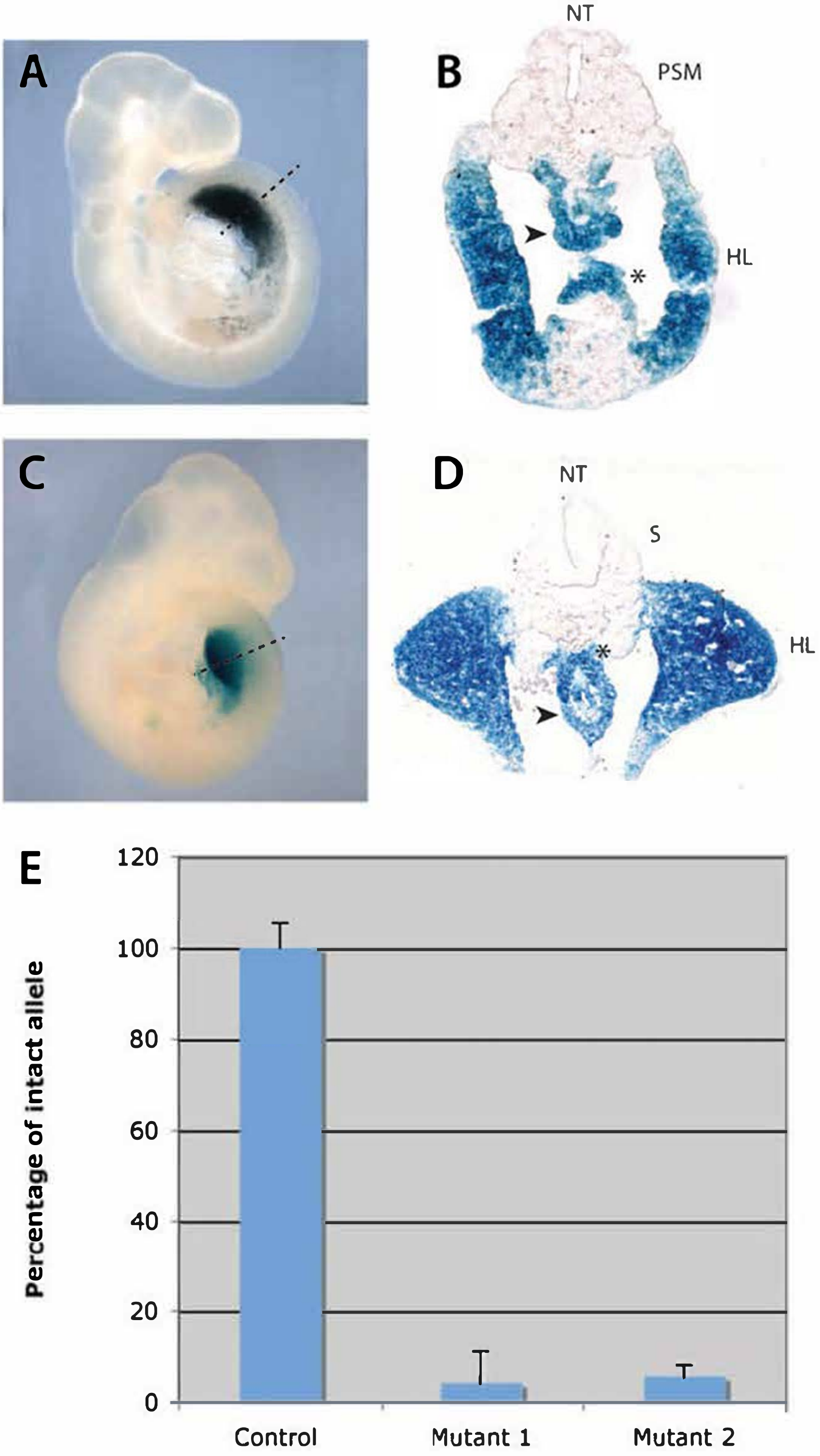

Development • Supplementary information

Fig. S1. Cre recombinase activity in the hindlimb-forming LPM in the *RetRV5Cre* transgenic deleter line. LacZ staining of Cre expressing cells at E9.75 (n=8). **(A,C)** right-side lateral views of whole embryos. **(B,D)** Transverse cryosections through the hindlimb regions of embryos. **A,** E9.5 *Rosa26RlacZ;RetRV5Cre* embryo. LacZ activity is detected in the nascent hindlimb bud. **B**, Transverse cryosection through the hindlimb region of an E9.5 *Rosa26RlacZ;RetRV5Cre* embryo. LacZ activity is additionally present in the hindgut diverticulum (black arrowhead) and anterior to the vitelline artery (black asterisk). **C**, E10.5 *Rosa26RlacZ;RetRV5Cre* embryo, LacZ activity is detectable throughout the hindlimb buds. **D**, Transverse cryosection through the hindlimbs of an E10.5 *Rosa26RlacZ;RetRV5Cre* embryo, in which LacZ activity is not detectable in the ectoderm. LacZ activity is visible in the mesoderm of the hindgut (black arrowhead) and the dorsal mesentery (black asterisk). NT–Neural tube, S–somite, PSM–presomitic mesoderm, HL–hindlimb **E**, Graphical representation of Q-PCR results to analyse the extent of deletion of the *Tbx4* conditional allele in 2 separate E10.5 $Tbx4^{lox/lox}$*;RetRV5Cre* hindlimbs . An estimated 96% of conditional *Tbx4* allele has been excised in $Tbx4^{lox/lox}$*;RetRV5Cre* mutant hindlimbs.

Development • Supplementary information

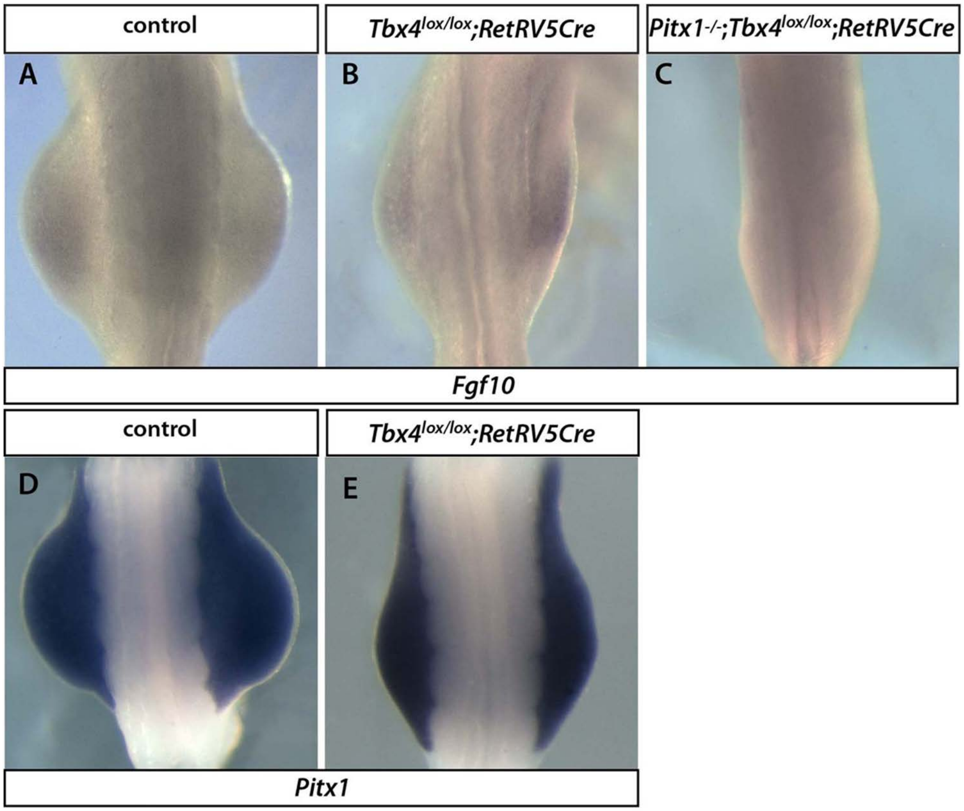

Fig. S2. *Fgf10* expression is regulated by dual inputs from *Tbx4* and *Pitx1*. Whole mount *in situ* hybridisation showing *Fgf10* expression in E10.25 hindlimbs of **A**, control (n=8), **B**, *Tbx4*$^{lox/lox}$;*RetRV5Cre* (n=3) **C**, *Pitx1*$^{-/-}$;*Tbx4*$^{lox/lox}$;*RetRV5Cre* (n=2) **D,E** *Pitx1*

Development • Supplementary information

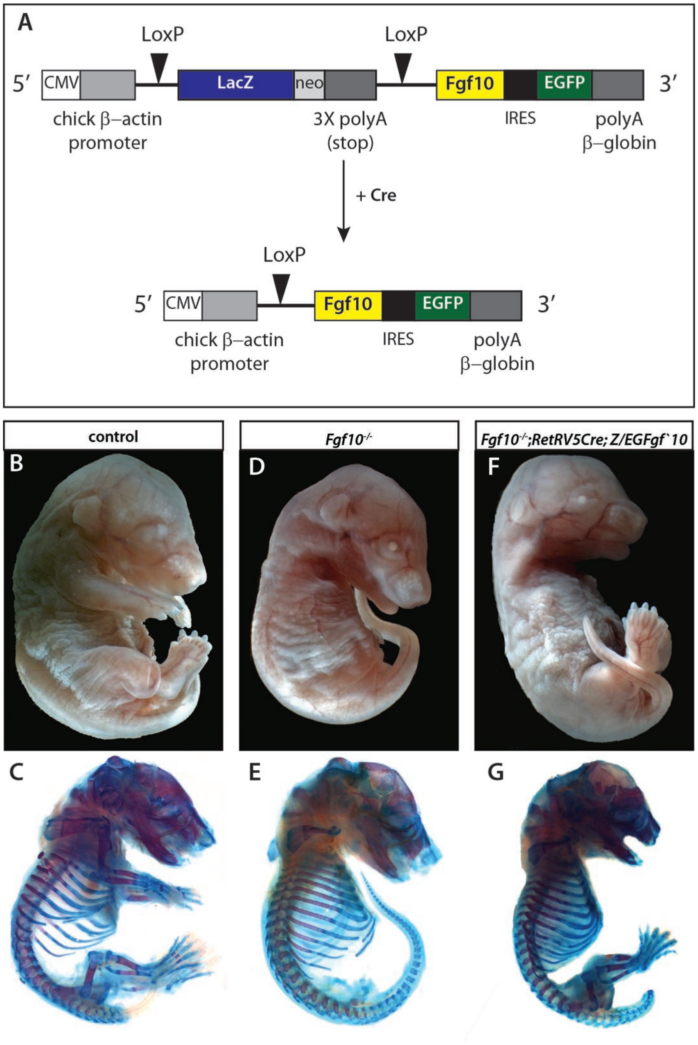

Suppl. Figure3

Development • Supplementary information

Fig. S3. The *Z/EGFgf10* transgene is able to fully rescue hindlimb outgrowth in *Fgf10*$^{-/-}$ embryos when activated by the *RetRV5Cre* line. A, Cre recombinase recombines the *LoxP* flanked cassette to enable transgenic *Fgf10* transcription in the *Z/EGFgf10* construct. The *Z/EGFgf10* construct was produced using the *Z/EG* transgenic backbone (Novak et al. 2000) and contains a β-Geo cassette, flanked by two *LoxP* sites (black triangles) downstream of the chick β-actin promoter (light grey rectangle) and the CMV enhancer (white rectangle). The β-Geo cassette contains *LacZ* sequence (blue rectangle) and a neomycin resistance gene (neo), as well as a 3x polyadenylation sites (dark grey) that serve as a transcriptional stop signal. Located 3' of the β -Geo cassette is the *Fgf10* cDNA (yellow rectangle) and an internal ribosome entry site (*IRES*, black rectangle), *eGFP* (green rectangle) and β-globin polyadenylation signal (dark grey rectangle). In the absence of Cre recombinase, CAGGS drives transcription of β-Geo. Following cre excision, the β-Geo cassette is removed and the *Fgf10*/*IRES*/*eGFP* cassette comes under the control of the CAGGS promoter. **B,D,F**, E17.5 embryos and **C,E,G**, complementary skeletal preparation of **B**,**C** control (n=1), **D,E** *Fgf10*$^{-/-}$ (n=1) and **F**,**G** *Fgf10*$^{-/-}$*;RetRV5Cre;Z/EGFgf10* rescued embryos (n=2). Hindlimb formation is completely rescued in the *Fgf10* mutant by activation of the *Fgf10* transgene.

Development • Supplementary information

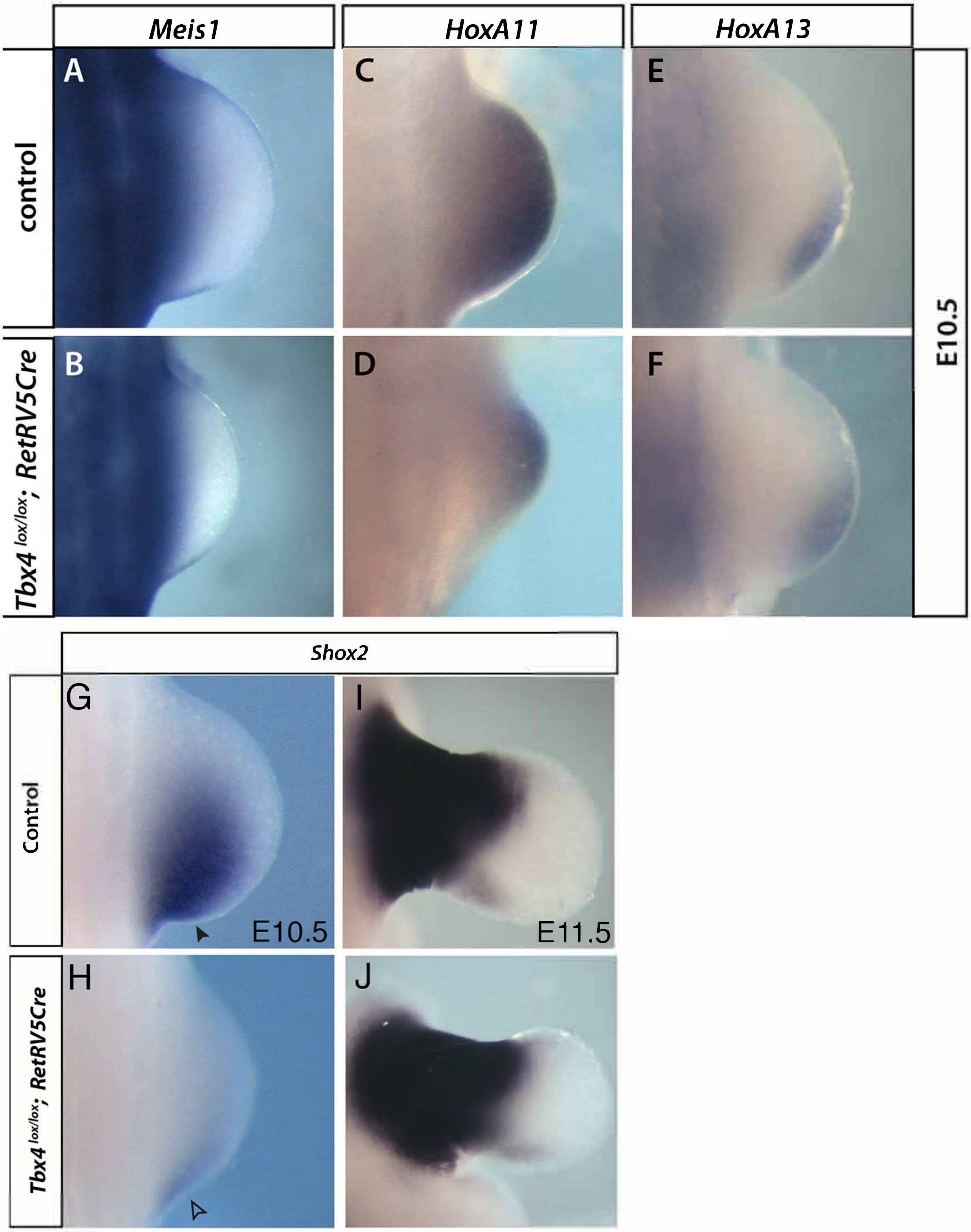

Development • Supplementary information

Fig. S4. *Meis1*, *Hoxa11* and *Hoxa13* are expressed in *Tbx4* mutant hindlimb buds. A-F Whole mount *in situ* hybridisation of E10.5 hindlimb buds. Dorsal views. Expression of the proximal segment (stylopod) marker *Meis1* (n=3) (**A**) is expressed in the *Tbx4*$^{lox/lox}$*;RetRV5Cre* mutant (n=3) (**B**). Medial segment (zeugopod) marker *Hoxa11* (n=3) (**C**) is expressed in the *Tbx4* mutant (n=2) (**D**). At E10.5 *Hoxa11* is expressed both medially and distally. Distal segment (autopod) marker *Hoxa13* (n=3) (**E**) is expressed in the *Tbx4* mutant (n=3) (**F**). ***Shox2* is downregulated in the *Tbx4* mutant at E10.5** Whole mount *in situ* hybridisation of E10.5 control (n=4) **(G)** and *Tbx4*$^{lox/lox}$*;RetRV5Cre* mutant (n=3) **(H)**. Robust expression in control (solid arrow) contrasts with reduced expression in the *Tbx4* conditional mutant (hollow arrow). *Shox2* expression is restricted to the proximal limb bud at E11.5 in control (n=3) (**I**) and *Tbx4*$^{lox/lox}$*;RetRV5Cre* mutant (n=2) (**G**).

Development • Supplementary information

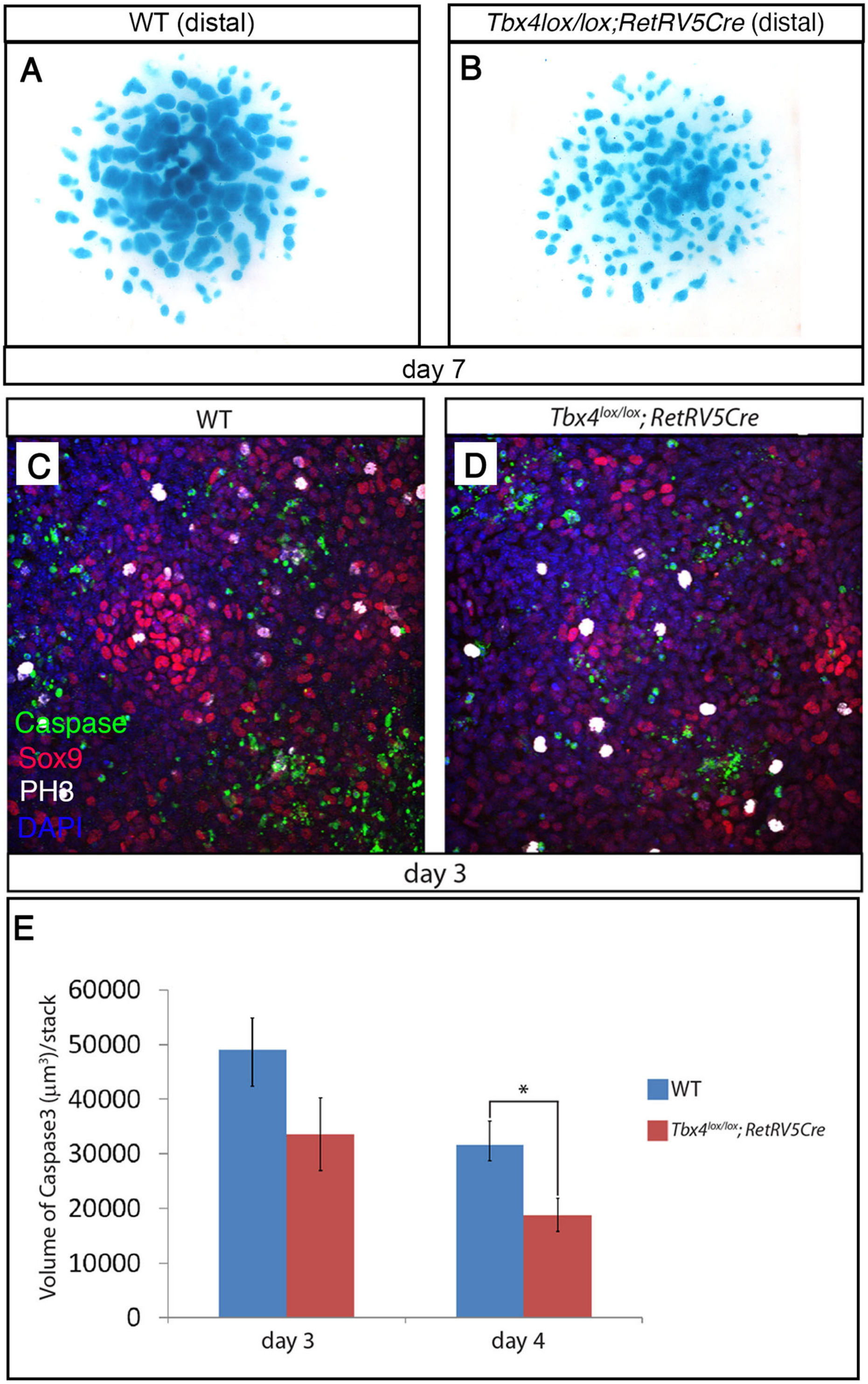

WT (distal)
Tbx4lox/lox;RetRV5Cre (distal)
A
B
day 7
WT
Tbx4lox/lox; RetRV5Cre
C
D
Caspase
Sox9
PH3
DAPI
day 3
E
Volume of Caspase3 (μm³)/stack
60000
50000
40000
30000
20000
10000
0
*
day 3
day 4
WT
Tbx4lox/lox; RetRV5Cre

Fig. S5. Cell death is not responsible for the decreased number of chondroprogenitors detected over time. A,B Alcian Blue staining of cartilage nodule formation after 7 days of culture in micromass cultures established from distal cells of **(A)** Wild type hindlimbs (n=6), **(B)** *Tbx4lox/lox;RetRV5Cre* hindlimbs (n=6). **C,D** Extended focus confocal stacks of immunostained proximal micromass culture after 3 days culture. Anti-Caspase (green), anti-Sox9 (red), phospho-histone H3 (white) and nuclear staining (DAPI-blue) of control (C) and *Tbx4*$^{lox/lox}$*;RetRV5Cre* (D) proximal micromass. **(E)** Histogram showing mean ± s.e.m. volume in μm^3 of Caspase 3 staining measured in a stack at 20x magnification of control (blue) and *Tbx4*$^{lox/lox}$*;RetRV5Cre* (red) proximal micromass after 3 and 4 days of culture. No statistical difference is observed between the two conditions after Student *t*-test (P>0.05); n=5. Standard errors are shown.

Development • Supplementary information

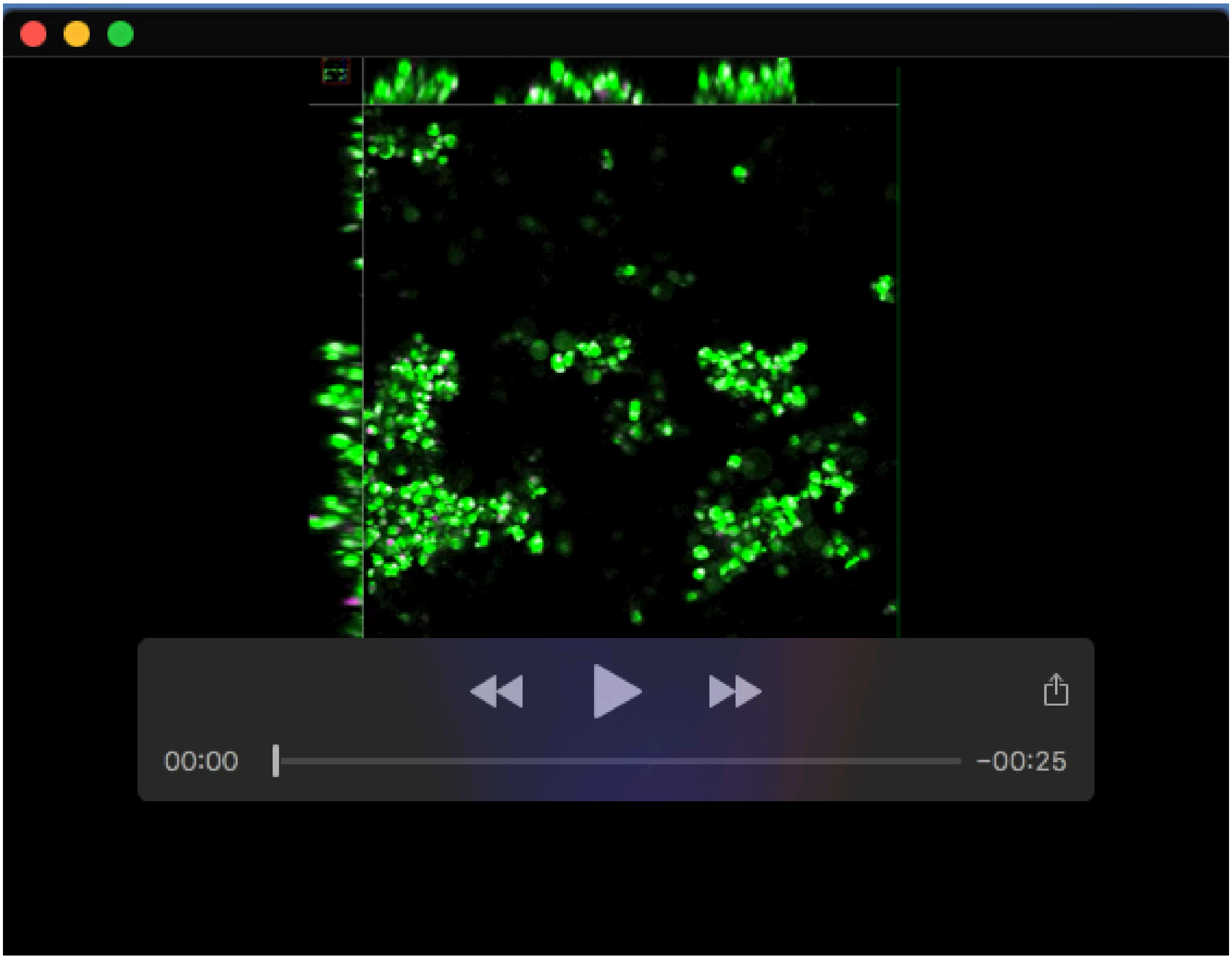

Movie 1. Time lapse movie of a control micromass culture stained with Cell Tracker dye. xyz views of Z-scan stacks imaged every 5 minutes during an 84hr culture. 20X magnification.

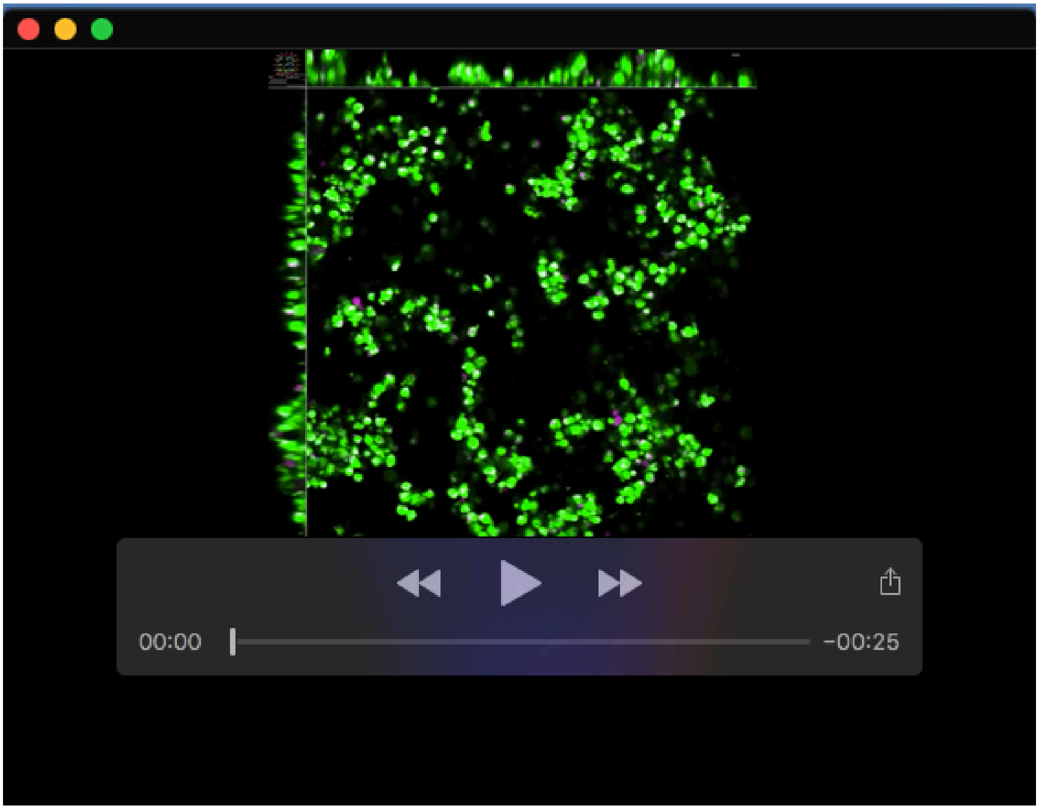

Movie 2. Time lapse movie of a *Tbx4*$^{lox/lox}$*;RetRV5Cre* micromass culture stained with Cell Tracker dye. xyz views of Z-scan stacks imaged every 5 minutes during an 84hr culture. 20X magnification.

Development • Supplementary information
