# Supplementary material for: Tbx4 function during hindlimb development reveals a mechanism that explains the origins of proximal limb defects
Source: Development. 2021 Sep 24;148(19):dev199580. doi: 10.1242/dev.199580 (PMC8497778; doi:10.1242/dev.199580)
Supplement: Reviewer comments [file develop-148-199580_review_history.pdf]

Tbx4 function during hindlimb development reveals a mechanism that explains the origins of proximal limb defects

Veronique Duboc, Fatima A. Sulaiman, Eleanor Feneck, Anna Kucharska, Donald Bell, Muriel Holder-Espinasse and Malcolm P. O. Logan
DOI: 10.1242/dev.199580

Editor: Liz Robertson

Review timeline

Original submission:	2 March 2021
Editorial decision:	6 April 2021
First revision received:	29 June 2021
Editorial decision:	27 July 2021
Second revision received:	29 July 2021
Accepted:	2 August 2021

Original submission

First decision letter

MS ID#: DEVELOP/2021/199580

MS TITLE: Tbx4 function during hindlimb development reveals a novel mechanism to explain the origins of proximal limb defects

AUTHORS: Veronique Duboc, Fatima A Sulaiman, Eleanor Feneck, Anna Kucharska, Donald Bell, and Malcolm Logan

I have now received all the referees reports on the above manuscript, and have reached a decision. The referees' comments are appended below, or you can access them online: please go to BenchPress and click on the 'Manuscripts with Decisions' queue in the Author Area.

The overall evaluation is positive and we would like to publish a revised manuscript in Development, provided that the referees' comments can be satisfactorily addressed. Please attend to all of the reviewers' comments in your revised manuscript and detail them in your point-by-point response. If you do not agree with any of their criticisms or suggestions explain clearly why this is so. As you will see Reviewer 1 is less enthusiastic about the work, but I found their review somewhat difficult to follow, and the English is not very clear. I think their criticisms in the main can be addressed in your Response to the Reviewers section.

We are aware that you may currently be unable to access the lab to undertake experimental revisions. If it would be helpful, we encourage you to contact us to discuss your revision in greater detail. Please send us a point-by-point response indicating where you are able to address concerns raised (either experimentally or by changes to the text) and where you will not be able to do so within the normal timeframe of a revision. We will then provide further guidance. Please also note that we are happy to extend revision timeframes as necessary.

Reviewer 1

Advance summary and potential significance to field

© 2021. Published by The Company of Biologists under the terms of the Creative Commons Attribution License (https://creativecommons.org/licenses/by/4.0/).

In this study Duboc et al., perform conditional mutants for Tbx4 by using a novel Cre line (RetRV5Cre) which removes Tbx4 earlier and completely from the HL as compared to previous studies. The resultant phenotype is a HL with reduced or missing proximal elements (pelvis, femur and fibula). By analyzing Fgf10 expression in these and compound Pitx1;Tbx4 mutants they propose that both genes are required for Fgf10 expression. They also analyze Islet1 and claim that it acts in parallel to Tbx4 and Pitx1 in hindlimb formation.
The authors showed that Tbx4lox/lox; RetRV5Cre phenotype can be rescued by both,Tbx4 and Tbx5. However, Fgf10 is not able to rescue this phenotype arguing that FGF signaling in the limb bud does not solely relay on Fgf10 regulation by Tbx4.
In order to find an explanation for the hypoplasia of proximal elements they analyzed genes involved in P-D patterning (Meis, Hoxa11 and Hoxa13) and Fgf8 and showed no change. They also discard the possibility that cell death causes the specific loss of the proximal elements showing no increase in cell death.
By micromass culture they study the possibility that these defects are caused by alteration in the chondrogenic process and found that in the proximal region chondroprogenitors fail to differentiate to chondrocytes. These cells do not maintain Sox9 expression and fail to express Sox5 and 6 (expressed during chondrogenic differentiation). Moreover, by studying cell shape and cell adhesion they propose that Tbx4 mutants do not show chondrogenic compaction and this would lead to the failure of proximal elements formation.

Comments for the author

While some results of the paper are interesting, it is unclear for the reviewer most of the conclusions Duboc et al., reached.

The argument for the use of a new Cre line is that previous lines did not delete Tbx4 from early HL development. However, there is no data demonstrating the timing and dynamics of Tbx4 deletion. Moreover, the presented phenotype is almost equal to that shown before for conditional Tbx4 mutants (L.A. Naiche et al.,2007 using ERCre and Prx1Cre). Therefore, the novelty of the phenotype is not clear.

The number of specimens should be presented for all the experiments in order to interpret the relevance of the results, and, if possible, quantifications. Mainly in those results in which slight expression reduction is shown.

All the conclusions regarding Fgf10 are not convincing as Fgf10 in situ is sub-standard (Supp Fig.2) and its normal expression is not even shown in the WT limbs, which at this stage should have strong Fgf10 expression. Therefore, all the conclusions rising from this result are not conclusive.

One of the main conclusions in the study is that different mechanisms act in proximal and distal regions. This finding is partially interpreted from a Col2a1 in situ in which slight reduction in Tbx4 mutants might simply be due to the difference in limb size. Even being smaller, there is still faint signal in the pelvis and femur. The result is also presented by Sox9 immunostaining and Col2-GFP, however, quality difference between control and mutant makes difficult the comparison and the lack of N number hard to conclude something.
The other experiment in which this conclusion relies is the micromass culture with distal tissue as compared to that with proximal tissue. However, in Supp Fig.5 it is shown that there is a reduction in Alcian Blue staining also in micromass culture from distal tissue. In the text it is mentioned that the reduction in staining is smaller, but lacking a quantification or number of experiments performed makes difficult to reach conclusions.
Even though it is not very well presented, it is convincing that there are problems in the compaction process in Tbx4 mutants (clear difference in micromass culture, lack of Sox5 and 6, different cell behavior). But it is not clear and explained the biased proximal defects.

Therefore, although the part of Tbx4 role in the chondrogenic process it is a novel finding and there are interesting experiments, the main conclusions of the article are not well supported and I would not recommend the paper for publication.

© 2021. Published by The Company of Biologists under the terms of the Creative Commons Attribution License (https://creativecommons.org/licenses/by/4.0/).

Reviewer 2

Advance summary and potential significance to field

In this study, Duboc et al. perform a very nice genetic analysis of the transcriptional network controlling hind limb initiation and outgrowth. Using conditional alleles for Tbx4 and Pitx1, combined with alleles allowing the targeted expression of Tbx5 or Fgf10, the authors convincingly show that Tbx4 and Pitx1 act in parallel and can in part compensate for each other, that Tbx5 can replace for Tbx4 in the hind limb, and that Isl1 acts in parallel with Tbx4/Pitx1, and cannot compensate loss of Tbx4/Pitx1. Moreover, it is shown that Tbx4 is required, specifically in hind limb proximal mesenchyme, for the maintenance of chondrogenic differentiation / fate. There are two especially compelling findings in this manuscript, that will be of high interest to the limb development community: a) the finding that an Fgf10 transgene does not rescue loss of Tbx4 is intriguing (it would be interesting to see if expression of Fgf10 rescues in part the phenotype of Tbx4/Pitx1 dko embryos, though), and b) that Tbx4 is required to an increased extent in proximal chondroprogenitors as opposed to distal ones. This is a very well executed study; the genetic data presented are compelling, congratulations.

Comments for the author

Comments:

1) I only see one issue the authors should address: the study of apoptosis in the limb buds (Fig.4 M-R) and especially in micromass cultures (Fig.S5 B, C) are somewhat weaker than the rest of the data in this manuscript. Data in Fig. 4 M-R might be quantified on serial optical sections if possible. Cleaved caspase staining in Fig. 4 M-R is not entirely convincing. Here, TUNEL staining might give more reliable results. Maybe lysotracker could be used alternatively, although I am not sure if this is applicable in this system.

2) In Fig. 6D-F I find it difficult to acknowledge the cell shape differences mentioned by the authors. A quantification of length/height ratio might help.

Minor comments:

Are there Tbx4 binding sites in Sox5, 6, 9 promoters / enhancers? There is a Tbx4 ChIP dataset (Karolak et al. 2021, Respiratory Research 22); performed in lung fibroblasts, but could nevertheless be checked.

It is very nicely shown that Sox5 and Sox6 protein expression is downregulated in Tbx4-cKO micromass cultures. It would be beautiful to see whole-mount ISH for the respective genes parallel to this.

Fig. 7M suggests that Sox9+ chondroprogenitors "fall back" to a fibroblast fate. This is an intriguing finding, supported by the whole-mount ISH and the micromass data. To make this even more convincing, fibroblast identity of the cells could be checked in micromass cultures e.g. by immunostaining for fibroblast markers (ER-TR7, vimentin, possibly alpha-smooth muscle actin).

Page 7 first paragraph: although I understand the authors' rationale, it is a bit confusing for the reader that Fig. 3F is mentioned here, before Fig 3 A-E are explained.

Page 8 first paragraph: “These results demonstrate that the level of FGF signalling in the hindlimb is not established solely through the direct regulation of Fgf10 ligand by Tbx4 and that other Tbx4 targets have critical roles in establishing FGF signalling levels sufficient for normal limb outgrowth.” Does this necessarily have to work via Fgf signalling (later in the discussion the authors discuss the possibility of Tbx4 acting via alternative mechanisms)?

Reviewer 3

Advance summary and potential significance to field

© 2021. Published by The Company of Biologists under the terms of the Creative Commons Attribution License (https://creativecommons.org/licenses/by/4.0/).

This manuscript focuses on Tbx4 function in the mouse hindlimb. To further explore the role of Tbx4 in the development of the hindlimb, the authors have generated a new deleter line (RetRV5Cre) that is active in the HL mesoderm before bud initiation. Removal of Tbx4 with this new Cre line results in phocomelia, in high contrast to the removal of Tbx5 in the FL. By mouse genetics the authors also show that Pitx1 and Tbx4 are conjointly required for correct Fgf10 expression and they also explore the epistatic relationship with Isl1.
Finally, using micromass culture experiments, they demonstrate that the proximal defect is due to a failure in the early differentiation of the chondrogenic progenitors.

A key strength of this study is that it is based on solid mouse genetics that confirm the equivalence between Tbx4 and Tbx5 in limb development, as previously suggested by the authors. The Fgf10 rescue experiments are also tremendously informative.

Overall, the authors have combined elegant genetic and molecular analyses to provide important and interesting insights into limb morphogenesis. The study is well performed and written and the conclusions clearly presented and based on the data provided.

Comments for the author

I am providing some suggestions, most of them minor, that I think may help improve the paper if adequately considered by the authors:
- The abstract doesn't seem to appropriately reflect this study. The use of life image is limited and the analysis of the relationship between cell behaviors and gene expression based on correlations. In my opinion the last paragraphs in the introduction capture much better this work.
- It will help readers if the authors could provide more information about the Tbx4;RetRV5Cre phenotype particularly regarding the digits. Can the identity/phalanx number of the remaining digits be distinguished. Is digit 1 present? This is important to appreciate any anterior-posterior defect in relation to the anterior loss of Fgf8.
Related to the point above, the absence of Fgf8 expression in the anterior half of the AER without noticeably impact in the shape of the bud (Fig. 4H-J) is impressive and difficult to explain. It would be interesting to know if another AER-Fgf, namely Fgf4, is upregulated and substituting for Fgf8 at least partially.
In normal micromass cultures, the acellular voids described by the authors correspond to accumulation of ECM- Maybe the authors could comment on this.
The discussion is rather long and a little disorganized in some parts. The section referring to Shox2 should either be removed or supported by a supp figure. Maybe the new proposed mechanism should be a separate section from the revision of the other so far proposed mechanisms to explain phocomelia.
Minor:
- Please, indicate in M&M the procedure to dissect proximal versus distal and whether the behavior of distal mutant cells was also analyzed.
- I couldn't find any explanation (neither the title) for SuppMovies 1 and 2. In the text they are referred to as
"from day 1 to early day 4".
- On page 17th, 2nd line: "The canonical Wnt pathway is also known to prevent? Chondrogenic differentiation
- Please, indicate the number of embryos analyzed, phenotypes, etc. throughout the manuscript-

---

First revision

<u>Author response to reviewers' comments</u>

Reviewer 1 Advance Summary and Potential Significance to Field:
In this study Duboc et al., perform conditional mutants for Tbx4 by using a novel Cre line (RetRV5Cre) which removes Tbx4 earlier and completely from the HL as compared to previous studies. The resultant phenotype is a HL with reduced or missing proximal elements (pelvis, femur

© 2021. Published by The Company of Biologists under the terms of the Creative Commons Attribution License (https://creativecommons.org/licenses/by/4.0/).

and fibula). By analyzing Fgf10 expression in these and compound Pitx1;Tbx4 mutants they propose that both genes are required for Fgf10 expression. They also analyze Islet1 and claim that it acts in parallel to Tbx4 and Pitx1 in hindlimb formation.
The authors showed that Tbx4lox/lox; RetRV5Cre phenotype can be rescued by both,Tbx4 and Tbx5. However, Fgf10 is not able to rescue this phenotype arguing that FGF signaling in the limb bud does not solely relay on Fgf10 regulation by Tbx4.
In order to find an explanation for the hypoplasia of proximal elements they analyzed genes involved in P-D patterning (Meis, Hoxa11 and Hoxa13) and Fgf8 and showed no change. They also discard the possibility that cell death causes the specific loss of the proximal elements showing no increase in cell death.
By micromass culture they study the possibility that these defects are caused by alteration in the chondrogenic process and found that in the proximal region chondroprogenitors fail to differentiate to chondrocytes. These cells do not maintain Sox9 expression and fail to express Sox5 and 6 (expressed during chondrogenic differentiation). Moreover, by studying cell shape and cell adhesion they propose that Tbx4 mutants do not show chondrogenic compaction and this would lead to the failure of proximal elements formation.

Reviewer 1 Comments for the Author:
While some results of the paper are interesting, it is unclear for the reviewer most of the conclusions Duboc et al., reached.

With respect, we strongly disagree with the reviewer on this point. We believe the conclusions we make are fully supported by the data we present. Our belief is supported by the other 2 reviewers of the manuscript.

The argument for the use of a new Cre line is that previous lines did not delete Tbx4 from early HL development. However, there is no data demonstrating the timing and dynamics of Tbx4 deletion. Moreover, the presented phenotype is almost equal to that shown before for conditional Tbx4 mutants (L.A. Naiche et al.,2007 using ERCre and Prx1Cre). Therefore, the novelty of the phenotype is not clear.

We do include data demonstrating the timing and dynamics pof the Tbx4 deletion. We include in Supplementary Figure 1 Cre recombinase activity in the hindlimb-forming LPM in the RetRV5Cre transgenic deleter line data that demonstrate the efficacy of the RetRvCre deleter transgenic using both a cre reporter ROSALacZ and also analysing recombination at the Tbx4 conditional allele. We conclude therefore that Tbx4 alleles will be deleted in the hindlimb-forming region prior to hindlimb bud formation and because this restricted expression does not produce chorioallantoic fusion defects it will bypass the early lethal phenotypes observed in previous studies.

The fact that the early deletion of Tbx4 produces a hindlimb phenotype that is similar to that previously reported using a cre delete that acts later than the one we use in this study is precisely the point we aim to make and this is an important, NOVEL aspect of our study. The point being that, even if Tbx4 is deleted prior to its activity in hindlimb forming regions, the hindlimb bud still forms and a smaller hindlimb develops, indicating, as we later investigate in this study, that Pitx1 can partially compensate in the absence of ANY Tbx4 activity. This was not clear from previous studies as the relatively late activity of the Prx1Cre means that it was not possible to rule out the possibility that some residual Tbx4 activity was present in the Tbx4 fl/fl Prx1Cre mutants originally presented.

The number of specimens should be presented for all the experiments in order to interpret the relevance of the results, and, if possible, quantifications. Mainly in those results in which slight expression reduction is shown.

The number of embryos analysed are now clearly indicated in the Materials and Methods section and respective Figure legends.
All the conclusions regarding Fgf10 are not convincing as Fgf10 in situ is sub-standard (Supp Fig.2) and its normal expression is not even shown in the WT limbs, which at this stage should have strong Fgf10 expression. Therefore, all the conclusions rising from this result are not conclusive.

© 2021. Published by The Company of Biologists under the terms of the Creative Commons Attribution License (https://creativecommons.org/licenses/by/4.0/).

We do not agree that the *Fgf10* in situ signal we present is sub-standard and this issue was not raised by the 2 other reviewers. In common with many other published studies, the signal produced by *Fgf10* RNA probes in whole mount *in situ* hybridisations is less robust when compared to probes for other gene transcripts.
Nevertheless, we believe our data is clear and entirely consistent with results published by other studies analysing *Fgf10* expression by whole mount *in situ* hybridisation. We include a control sample (panel A in Supp. Figure 2) as reference. Since the normal expression of Fgf10 is well documented the key comparison in this figure is actually the signal detected in the *Tbx4*$^{lox/lox}$*;RetRVCre* sample and the absence of signal in the *Pitx1*$^{-/-}$*;Tbx4*$^{lox/lox}$*;RetRVCre* sample.

One of the main conclusions in the study is that different mechanisms act in proximal and distal regions. This finding is partially interpreted from a Col2a1 in situ in which slight reduction in Tbx4 mutants might simply be due to the difference in limb size. Even being smaller, there is still faint signal in the pelvis and femur. The result is also presented by Sox9 immunostaining and Col2-GFP, however, quality difference between control and mutant makes difficult the comparison and the lack of N number hard to conclude something.
The other experiment in which this conclusion relies is the micromass culture with distal tissue as compared to that with proximal tissue. However, in Supp Fig.5 it is shown that there is a reduction in Alcian Blue staining also in micromass culture from distal tissue. In the text it is mentioned that the reduction in staining is smaller, but lacking a quantification or number of experiments performed makes difficult to reach conclusions.
Even though it is not very well presented, it is convincing that there are problems in the compaction process in Tbx4 mutants (clear difference in micromass culture, lack of Sox5 and 6, different cell behavior). But it is not clear and explained the biased proximal defects.

We show several lines of evidence to support, as we state in the text, that “in the absence of Tbx4, chondroprogenitors located in the proximal part of the limb bud fail to differentiate into chondrocytes” as an explanation for the proximal bias to the skeletal abnormalities seen in the Tbx4 mutants.
We show reduction/absence of a proximal domain by Col2a1 by in situ and reduction using a Col2GFP reporter. It is not practical to attempt to quantify the 3D domain of whole mount stain which is why we carried out further studies using micromass which we also include in Figure 6. WE have also included numbers of samples processed.
Fig6G is quantification of Sox9 staining. Staining of Sox5, Sox6 and PNA provide further support for the conclusions drawn. Overall, as the reviewer agrees, the data provided do show compaction issues in the *Tbx4* mutants to support the model we propose in the manuscript. We cannot yet fully explain the origins of the proximal defects, and do not claim to in our text, but rather this paper serves to demonstrate that proximal-distal differences do exist and are related to the activity of *Tbx4*.

Reviewer 2 Advance Summary and Potential Significance to Field:
In this study, Duboc et al. perform a very nice genetic analysis of the transcriptional network controlling hind limb initiation and outgrowth. Using conditional alleles for Tbx4 and Pitx1, combined with alleles allowing the targeted expression of Tbx5 or Fgf10, the authors convincingly show that Tbx4 and Pitx1 act in parallel and can in part compensate for each other, that Tbx5 can replace for Tbx4 in the hind limb, and that Isl1 acts in parallel with Tbx4/Pitx1, and cannot compensate loss of Tbx4/Pitx1. Moreover, it is shown that Tbx4 is required, specifically in hind limb proximal mesenchyme, for the maintenance of chondrogenic differentiation / fate. There are two especially compelling findings in this manuscript, that will be of high interest to the limb development community:
a) the finding that an Fgf10 transgene does not rescue loss of Tbx4 is intriguing (it would be interesting to see if expression of Fgf10 rescues in part the phenotype of Tbx4/Pitx1 dko embryos, though), and b) that Tbx4 is required to an increased extent in proximal chondroprogenitors as opposed to distal ones. This is a very well executed study; the genetic data presented are compelling, congratulations.
We thank the reviewers for highlighting the novel findings our data contribute to the community.

© 2021. Published by The Company of Biologists under the terms of the Creative Commons Attribution License (https://creativecommons.org/licenses/by/4.0/).

Reviewer 2 Comments for the Author:

1) I only see one issue the authors should address: the study of apoptosis in the limb buds (Fig.4 M-R) and especially in micromass cultures (Fig.S5 B, C) are somewhat weaker than the rest of the data in this manuscript. Data in Fig. 4 M-R might be quantified on serial optical sections if possible. Cleaved caspase staining in Fig. 4 M- R is not entirely convincing. Here, TUNEL staining might give more reliable results. Maybe lysotracker could be used alternatively, although I am not sure if this is applicable in this system.

We have modified the text describing the results using Lysotracker Red as an indicator of cell death in whole mount, shown in Figure4M-R.
We have modified Supplementary Figure 5 to clarify the results and quantification of Caspase staining in micromass, The key result is that there is no increase in cell death markers Caspase staining in the Tbx4 mutant limbs and micomass cultures.

2) In Fig. 6D-F I find it difficult to acknowledge the cell shape differences mentioned by the authors. A quantification of length/height ratio might help.

We have removed the text that discussed cell shape differences.

Minor comments:

Are there Tbx4 binding sites in Sox5, 6, 9 promoters / enhancers? There is a Tbx4 ChIP dataset (Karolak et al. 2021, Respiratory Research 22); performed in lung fibroblasts, but could nevertheless be checked.

None are described in the published dataset using lung fibroblasts. The ideal experiment would be to do ChIP seq or equivalent on limb bud material with antibodies for Tbx4. This would be interesting to do this but beyond the scope of current study. We are not aware of any published dataset that could be analysed.

It is very nicely shown that Sox5 and Sox6 protein expression is downregulated in Tbx4-cKO micromass cultures. It would be beautiful to see whole-mount ISH for the respective genes parallel to this.

Unfortunately, we do not have *in situ* data of Sox5/Sox6 mRNA transcript expression. We felt that analysis of the protein expression was sufficient to demonstrate the effect on Sox5/6 expression. It would require significant time and resources to obtain this additional data and we do not feel it is necessary for the conclusions we make in our manuscript.

Fig. 7M suggests that Sox9+ chondroprogenitors "fall back" to a fibroblast fate. This is an intriguing finding, supported by the whole-mount ISH and the micromass data. To make this even more convincing, fibroblast identity of the cells could be checked in micromass cultures e.g. by immunostaining for fibroblast markers (ER- TR7, vimentin, possibly alpha-smooth muscle actin).

We agree that this would be an interesting result to follow up in the future with fibroblast markers, however, to do this now will require extensive additional resources. We do not make any direct claims about the fate of chondroprogenitors in our text

Page 7 first paragraph: although I understand the authors' rationale, it is a bit confusing for the reader that Fig. 3F is mentioned here, before Fig 3 A-E are explained.

We have modified Figure 3 and we now place the schematic as panel 3A and the panels originally Fig3A-E are now Fig3B-F in line with the reviewer's suggestion.

Page 8 first paragraph: "These results demonstrate that the level of FGF signalling in the hindlimb is not established solely through the direct regulation of Fgf10 ligand by Tbx4 and that other Tbx4 targets have critical roles in establishing FGF signalling levels sufficient for normal limb outgrowth." Does this necessarily have to work via Fgf signalling (later in the discussion the authors

© 2021. Published by The Company of Biologists under the terms of the Creative Commons Attribution License (https://creativecommons.org/licenses/by/4.0/).

discuss the possibility of Tbx4 acting via alternative mechanisms)?

This statement relates to *Tbx4* activity during hindlimb initiation to establish the appropriate levels of Fgf10 and possibly Fgf receptor expression, similar to what we have reported for Tbx5 as we state in the text.

Reviewer 3 Advance Summary and Potential Significance to Field:
This manuscript focuses on Tbx4 function in the mouse hindlimb. To further explore the role of Tbx4 in the development of the hindlimb, the authors have generated a new deleter line (RetRV5Cre) that is active in the HL mesoderm before bud initiation. Removal of Tbx4 with this new Cre line results in phocomelia, in high contrast to the removal of Tbx5 in the FL. By mouse genetics the authors also show that Pitx1 and Tbx4 are conjointly required for correct Fgf10 expression and they also explore the epistatic relationship with Isl1.
Finally, using micromass culture experiments, they demonstrate that the proximal defect is due to a failure in the early differentiation of the chondrogenic progenitors.

A key strength of this study is that it is based on solid mouse genetics that confirm the equivalence between Tbx4 and Tbx5 in limb development, as previously suggested by the authors. The Fgf10 rescue experiments are also tremendously informative.

We thank the reviewer for highlighting the novelty of our mouse line and the knowledge gained from experiments carried out on this mouse knockout model.
Overall, the authors have combined elegant genetic and molecular analyses to provide important and interesting insights into limb morphogenesis. The study is well performed and written and the conclusions clearly presented and based on the data provided.

Reviewer 3 Comments for the Author:
I am providing some suggestions, most of them minor, that I think may help improve the paper if adequately considered by the authors:

- The abstract doesn't seem to appropriately reflect this study. The use of life image is limited and the analysis of the relationship between cell behaviors and gene expression based on correlations. In my opinion, the last paragraphs in the introduction capture much better this work.

We have taken on board this comment and have changed this section of the abstract as suggested by the reviewer.

- It will help readers if the authors could provide more information about the Tbx4;RetRV5Cre phenotype, particularly regarding the digits. Can the identity/phalanx number of the remaining digits be distinguished. Is digit 1 present? This is important to appreciate any anterior-posterior defect in relation to the anterior loss of Fgf8.

The skeletal phenotype of the mouse models are shown in Figure 3, where a single digit is lost in the Tbx4;RetRV5Cre. The identity of each digit in the mutant cannot be unequivocally identified. However, the most anterior digit that does remain has a morphology most like that of digit 1, we therefore propose that intermediate digits are lost.

We have modified the text in our revised manuscript to address this comment.

Related to the point above, the absence of Fgf8 expression in the anterior half of the AER without noticeably impact in the shape of the bud (Fig. 4H-J) is impressive and difficult to explain. It would be interesting to know if another AER-Fgf, namely Fgf4, is upregulated and substituting for Fgf8 at least partially.

Yes, we were also struck by this dramatic effect. We did not analyse expression of another AER-Fgf in these samples, however. Our impression was that the (smaller) bud that forms is derived from the posterior portion of the early, emergent bud and that the anterior cells do not proliferate due to the lack of AER-Fgf ligand.
In normal micromass cultures, the acellular voids described by the authors correspond to accumulation of ECM- Maybe the authors could comment on this.

© 2021. Published by The Company of Biologists under the terms of the Creative Commons Attribution License (https://creativecommons.org/licenses/by/4.0/).

We have edited the manuscript text to include a statement related to this comment:

"In normal micromass culture, the appearance of an acellular void corresponds to the accumulation of extracellular matrix. The absence of acellular voids in the *Tbx4* mutant micromass cultures could therefore indicate a failure of, or reduction in, the production of ECM."

The discussion is rather long and a little disorganized in some parts. The section referring to Shox2 should either be removed or supported by a supp figure. Maybe the new proposed mechanism should be a separate section from the revision of the other so far proposed mechanisms to explain phocomelia.

We have made changes to the Discussion section. We now include the Shox2 data in Supp. Figure 4 G-J.

Minor:

- Please, indicate in M&M the procedure to dissect proximal versus distal and whether the behavior of distal mutant cells was also analyzed.

We have indicated in the Materials and Methods the procedure used to generate proximal and distal cultures. "Hindlimbs were harvested from 11.5 d.p.c. *Tbx4*$^{lox/lox}$*;RetRV5Cre* conditional mutants and wild-type embryos. Hindlimb buds dissected from the flank of the embryo were bisected, transversely at the approximate proximal-distal midpoint to generate proximal and distal portions that were processed separately. Limbs and limb portions were dissociated in 1 unit/ml of dispase II (Roche Diagnostics) solution containing 10% fetal bovine serum (FBS)/Puck's saline A buffer for 20min at 37 °C."

- I couldn't find any explanation (neither the title) for SuppMovies 1 and 2. In the text they are referred to as "from day 1 to early day 4".

A description of the supplementary movies is now included at the end of the Figure legends section.

- On page 17th, 2nd line: "The canonical Wnt pathway is also known to prevent? Chondrogenic differentiation

The canonical Wnt pathway can affect chondrogenic differentiation. Following ectopic Wnt expression in chick micromass culture chondrocytes undergo compaction but their differentiation into chondroblasts is blocked (Day et al. 2005, Rudnicki and Brown 1997). Furthermore, conditional deletion of D-catenin in the mouse results in increased Sox9 expression and an increase in the number of chondrocytes, at the expense of osteoblasts (Day et al. 2005).

- Please, indicate the number of embryos analyzed, phenotypes, etc. throughout the manuscript-

The number of embryos analysed are now clearly indicated in the Materials and Methods section and respective Figure legends.

Second decision letter

MS ID#: DEVELOP/2021/199580

MS TITLE: Tbx4 function during hindlimb development reveals a novel mechanism to explain the origins of proximal limb defects

AUTHORS: Veronique Duboc, Fatima A Sulaiman, Eleanor Feneck, Anna Kucharska, Donald Bell, Muriel Holder-Espinasse, and Malcolm Logan

© 2021. Published by The Company of Biologists under the terms of the Creative Commons Attribution License (https://creativecommons.org/licenses/by/4.0/).

I have now received all the referees reports on the above manuscript, and have reached a decision. The referees' comments are appended below, or you can access them online: please go to BenchPress and click on the 'Manuscripts with Decisions' queue in the Author Area.

The overall evaluation is positive and we would like to publish a revised manuscript in Development, provided that the referees' very minor comments can be satisfactorily addressed. Please attend to all of the reviewers' comments in your revised manuscript and detail them in your point-by-point response. If you do not agree with any of their criticisms or suggestions explain clearly why this is so.

We are aware that you may currently be unable to access the lab to undertake experimental revisions. If it would be helpful, we encourage you to contact us to discuss your revision in greater detail. Please send us a point-by-point response indicating where you are able to address concerns raised (either experimentally or by changes to the text) and where you will not be able to do so within the normal timeframe of a revision. We will then provide further guidance. Please also note that we are happy to extend revision timeframes as necessary.

Reviewer 1

Advance summary and potential significance to field

After rivision authors have answered what was suggested and, upon resolution of two essential not-answered points, I would propose the article for publication.

Comments for the author

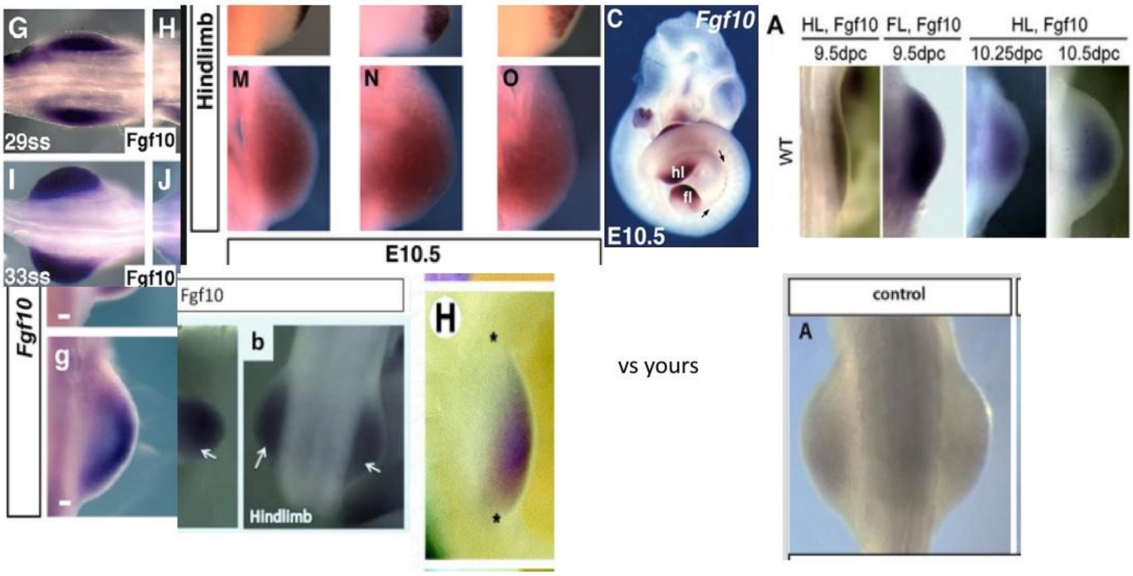

References:
-BMP signals control limb interdigital programmed cell death by regulating FGF signaling. Fig.2
-*Bmp2*, *Bmp4* and *Bmp7* Are Co-Required in the Mouse AER for Normal Digit Patterning but Not Limb Outgrowth. Fig.3
-Role of FGF10/FGFR2b signaling during mammary gland development in the mouse embryo . Fig.3
-LIM homeobox transcription factors integrate signaling events that control three-dimensional limb patterning and growth. Fig5
-Control of mouse limb initiation and antero-posterior patterning by Meis transcription factors. Fig.4
-Isl1 and Ldb co-regulators of transcription are essential early determinants of mouse limb development. Fig.3
-Mouse *Twist* is required for fibroblast growth factor-mediated epithelial–mesenchymal signalling and cell survival during limb morphogenesis. Fig.3

© 2021. Published by The Company of Biologists under the terms of the Creative Commons Attribution License (https://creativecommons.org/licenses/by/4.0/).

Reviewer 2

Advance summary and potential significance to field

Thank you for addressing my comments; referring to your note "We have modified Supplementary Figure 5 to clarify the results and quantification of Caspase staining in micromass": to me the figure appears identical to the previous version...?
Side note: the figure labels are wrong or absent; regarding the figure legend, micromass images should be labelled "A" and "B", immunostaining images should be "C" and "D", histogram should be "E".

Comments for the author

see above

Reviewer 3

Advance summary and potential significance to field

This manuscript focuses on Tbx4 function in the mouse hindlimb. To further explore the role of Tbx4 in the development of the hindlimb, the authors have generated a new deleter line (RetRV5Cre) that is active in the HL mesoderm before bud initiation. Removal of Tbx4 with this new Cre line results in phocomelia, in high contrast to the removal of Tbx5 in the FL. By mouse genetics the authors also show that Pitx1 and Tbx4 are conjointly required for correct Fgf10 expression and they also explore the epistatic relationship with Isl1.
Finally, using micromass culture experiments, they demonstrate that the proximal defect is due to a failure in the early differentiation of the chondrogenic progenitors.

Comments for the author

The authors have appropriately responded to my comments. I have no further request.

Second revision

Author response to reviewers' comments

We had incorrectly incldued an old version of Supplementary Figure 5 in our previous revision. We have now corrected this and have incorporated a new Supplementary Figure 5 that addresses the original comments from the reviewer.

Third decision letter

MS ID#: DEVELOP/2021/199580

MS TITLE: Tbx4 function during hindlimb development reveals a novel mechanism to explain the origins of proximal limb defects

AUTHORS: Veronique Duboc, Fatima A Sulaiman, Eleanor Feneck, Anna Kucharska, Donald Bell, Muriel Holder-Espinasse, and Malcolm Logan
ARTICLE TYPE: Research Article

I am happy to tell you that your manuscript has been accepted for publication in Development, pending our standard ethics checks.

© 2021. Published by The Company of Biologists under the terms of the Creative Commons Attribution License (https://creativecommons.org/licenses/by/4.0/).
